# Calprotectin elicits aberrant iron starvation responses in *Pseudomonas aeruginosa* under anaerobic conditions

**DOI:** 10.1128/jb.00029-25

**Published:** 2025-03-26

**Authors:** Jacob M. Weiner, Wei Hao Lee, Elizabeth M. Nolan, Amanda G. Oglesby

**Affiliations:** 1 Department of Pharmaceutical Sciences, School of Pharmacy, University of Maryland 15513 , Baltimore, Maryland, USA; 2 Department of Chemistry, Massachusetts Institute of Technology 201648 , Cambridge, Massachusetts, USA; 3 Department of Microbiology and Immunology, School of Medicine, University of Maryland 12264 , Baltimore, Maryland, USA; Geisel School of Medicine at Dartmouth, Hanover, New Hampshire, USA

**Keywords:** iron, calprotectin, metals, iron regulation, *Pseudomonas aeruginosa*, PrrF

## Abstract

*Pseudomonas aeruginosa* is an opportunistic pathogen that uses several mechanisms to survive in the iron-limiting host environment. The innate immune protein calprotectin (CP) sequesters ferrous iron [Fe(II)], among other divalent transition metal ions, to limit its availability to pathogens. CP levels are increased in individuals with cystic fibrosis (CF), a hereditary disease that leads to chronic pulmonary infection by *P. aeruginosa*. We previously showed that aerobic CP treatment of *P. aeruginosa* induces a multi-metal starvation response that alters expression of several virulence properties. However, the CF lung is a hypoxic environment due to the growth of *P. aeruginosa* in dense biofilms. Here, we report that anaerobic CP treatment of *P. aeruginosa* induces many processes associated with an aerobic iron starvation response, including decreased phenazine production and increased expression of the PrrF small regulatory RNAs (sRNAs). However, the iron starvation response elicited by CP in anaerobic conditions shows characteristics that are distinct from responses observed in aerobic growth, including a lack of siderophore production and increased induction of genes for the FeoAB Fe(II) and Phu heme uptake systems. Also distinct from aerobic conditions, CP treatment induces expression of genes for predicted manganese transporters MntH1 and MntH2 during anaerobic growth while eliciting a less robust zinc starvation response compared to aerobic conditions. Induction of *mntH2* is dependent on the PrrF sRNAs, suggesting a novel example of metal regulatory cross-talk. Thus, anaerobic CP treatment results in a multi-metal starvation response with key distinctions from aerobic conditions, revealing differences in *P. aeruginosa* metal homeostasis during anaerobic growth.

**IMPORTANCE:**

Iron is critical for most microbial pathogens, and the innate immune system sequesters this metal to limit microbial growth. Pathogens must overcome iron sequestration to survive during infection. For many pathogens, iron homeostasis has primarily been studied in aerobic conditions. Nevertheless, some host environments are hypoxic, including chronic lung infection sites in individuals with cystic fibrosis (CF). Here, we use the innate immune protein calprotectin, which sequesters divalent metal ions including Fe(II), to study the anaerobic iron starvation response of a common CF lung pathogen, *Pseudomonas aeruginosa*. We report several distinctions of this response during anaerobiosis, highlighting the importance of carefully considering the host environment when investigating the role of nutritional immunity in host-pathogen interactions.

## INTRODUCTION

*Pseudomonas aeruginosa* is a gram-negative multi-drug-resistant opportunistic pathogen that causes both acute and chronic infections in vulnerable patients. *P. aeruginosa* can invade and persist in multiple locations within the body, leading to disease states including pneumonia, endocarditis, keratitis, meningitis, and septicemia (1–7). These infections are challenging to combat due to various mechanisms of intrinsic and acquired drug resistance (8–10). Additionally, formation of biofilms in chronic and device-mediated infections results in increased antibiotic tolerance and escape from the immune system (10). *P. aeruginosa* is one of the most prevalent microorganisms in the lungs of patients with cystic fibrosis (CF), a hereditary disease that results in the accumulation of thick dehydrated mucus in the airways and persistent polymicrobial lung infections (5, 11–15). Carriage of *P. aeruginosa* in the CF lung is associated with the presence of hypoxic, biofilm-containing mucus plugs, resulting in worsened lung function and eventually patient mortality (11, 16, 17). Understanding how *P. aeruginosa* survives in this hypoxic environment is vital to developing effective therapeutics for CF lung infections.

One avenue of therapeutic interest is targeting nutrient uptake systems to either inhibit *P. aeruginosa* growth or enhance delivery of current antimicrobials into the cell (18–23). *P. aeruginosa* requires transition metals, most notably iron, for use as cofactors in various enzymes (24–26). To prevent microbial growth, the host immune system produces several transition metal-sequestering proteins in a process termed nutritional immunity (26–28). *P. aeruginosa* attempts to overcome this host response by deploying numerous iron uptake systems (25, 29, 30). These systems include the secretion of the siderophores pyoverdine and pyochelin, secondary metabolites that scavenge Fe(III) and are then transported to the cytoplasm through specific receptors and transport machinery (31). *P. aeruginosa* also acquires Fe(II), which is more prevalent in reducing and anaerobic environments, via the FeoAB system (32, 33). Additionally, *P. aeruginosa* can acquire host heme through two non-redundant heme uptake systems—the heme assimilation system (Has) and the *Pseudomonas* heme uptake system (Phu) (34, 35). Internalized heme is then degraded via heme oxygenase to release iron (36). Together, these systems allow *P. aeruginosa* to acquire iron in various conditions, contributing to its persistence during infection.

Iron uptake must be tightly regulated in response to intracellular iron concentration, as too much iron results in oxidative stress while too little iron hinders critical metabolic functions. Iron regulation in *P. aeruginosa* is largely controlled by the ferric uptake regulator (Fur) protein (37, 38). When iron is abundant within the cell, Fur binds to Fe(II), thus increasing its affinity for promoters to block the expression of various genes including those encoding iron uptake systems (37, 38). When cytosolic iron is scarce, the apo-Fur protein is inactive, leading to de-repression of its target genes (37). Fur also represses the PrrF1 and PrrF2 small regulatory RNAs (sRNAs), which are expressed upon iron starvation and block the translation of mRNAs encoding nonessential iron-cofactored proteins in a process termed the iron-sparing response (39, 40). The PrrF sRNAs also indirectly promote the production of the *Pseudomonas* quinolone signal (PQS), which co-activates the expression of several virulence genes (41–43). This occurs via repression of the AntR regulator that activates expression of genes for degradation of the anthranilate, the precursor of PQS (44). Due to the tandem arrangement of the *prrF1* and *prrF2* sRNAs, *P. aeruginosa* produces a third heme-responsive sRNA, named PrrH, made from transcriptional read-through of the *prrF1* terminator, allowing transcription to extend through the *prrF2* sequence (45, 46). The PrrH sRNA was recently shown to block expression of genes for pyochelin production, suggesting a role for heme regulatory cross-talk with ferric siderophore uptake systems (45). Together, these regulatory elements allow for a delicate balance of iron inventory within *P. aeruginosa* cells.

Many studies have addressed how the host limits iron availability in aerobic environments where the Fe(III) oxidation state is favored, such as the production of proteins that bind to Fe(III) and prevent its acquisition by pathogens. These proteins include lactoferrin, which sequesters Fe(III), and siderocalin (also known as lipocalin-2 or NGAL), which captures Fe(III)-bound siderophores (26, 27). Less is known about how the host sequesters Fe(II), which is a relevant oxidation state in biology, particularly in hypoxic environments. Moreover, while some studies have investigated *P. aeruginosa* iron uptake systems that function during hypoxia, such as FeoAB, far less is known about how *P. aeruginosa* mediates iron regulation in low oxygen environments. Two studies evaluating the effects of *P. aeruginosa* Fur on virulence gene expression showed decreased production of pyoverdine and the Fur-regulated exotoxin A under microaerobic conditions compared to aerobic conditions (47, 48). Despite the finding that iron regulation is altered under microaerobic conditions, a thorough analysis of how oxygen impacts *P. aeruginosa* iron regulation has not been performed.

Previous studies of the innate immune protein human calprotectin (CP) show it sequesters Fe(II) and elicits iron starvation responses in multiple bacterial pathogens, including *P. aeruginosa,* under aerobic conditions (49–55). CP is a heterooligomer of calcium-binding proteins S100A8 (calgranulin A, MRP8) and S100A9 (calgranulin B, MRP14) (56–58). Each heterodimer has two Ca(II)-binding sites and two transition metal-binding sites, and Ca(II) binding to the heterodimer causes self-association to form a heterotetramer that displays enhanced metal-binding affinities, growth inhibitory activity, and protease resistance (57–60). Thus, the working model shows that CP exists as a heterodimer in the neutrophil cytoplasm where Ca(II) levels are low and as a Ca(II)-bound heterotetramer following release from the cell into the extracellular space where Ca(II) levels are high (58, 59, 61). The two sites for transition metal binding differ: site 1 (His_3_Asp) sequesters Zn(II), and site 2 (His_6_) is functionally versatile and sequesters multiple nutrient metal ions, including Mn(II), Fe(II), Ni(II), and Zn(II) (49, 58, 59, 62–65). Under aerobic conditions, CP has antimicrobial activity against a variety of bacterial pathogens due to its ability to sequester transition metals (49, 50, 66). For *P. aeruginosa*, the ability of CP to inhibit its growth under aerobic conditions is attributed to iron sequestration, but CP treatment also induces a robust Zn starvation response in addition to multi-metal starvation responses in this pathogen (49, 51, 53). By contrast, there has been limited work evaluating how CP influences growth and metal homeostasis for bacterial pathogens cultured under low oxygen conditions (55, 67), which is relevant to many disease states. Because CP binds to Fe(II), and because hypoxia alters iron regulatory pathways, we reasoned that the impacts of CP on *P. aeruginosa* metal homeostasis will be altered under anaerobic conditions.

In this study, we investigated how CP treatment influences *P. aeruginosa* iron starvation responses and global gene expression under anaerobic conditions. We show that anaerobic CP treatment induces an overlapping yet distinct iron starvation response from that previously characterized in aerobic conditions. For example, anaerobic CP treatment results in a robust increase in both the *feo* Fe(II) uptake and *phuR* heme uptake genes, neither of which are induced by aerobic CP treatment (51, 53, 54). Compared to aerobic conditions, anaerobic CP treatment resulted in an attenuated zinc starvation response and an apparent manganese starvation response, suggesting different metal requirements during anaerobic growth. Moreover, CP-mediated induction of *mntH2*, encoding a putative Mn uptake protein, and *sodA*, encoding the manganese-cofactored superoxide dismutase, is dependent upon the PrrF sRNAs during anaerobic growth. Overall, this work provides the first comprehensive analysis of how *P. aeruginosa* responds to CP-mediated metal sequestration during anaerobic growth, which is a critical step for understanding host-pathogen interactions in chronic infections where biofilms and hypoxia are prevalent.

## RESULTS

### CP exhibits antimicrobial activity against *P. aeruginosa* under anaerobic conditions

The ability of CP to inhibit growth of *P. aeruginosa* under aerobic conditions is dependent on its ability to sequester Fe(II) (49, 51). To determine whether CP inhibits *P. aeruginosa* growth under anaerobic conditions, we first optimized a previously described anaerobic growth system (68, 69). *P. aeruginosa* strain PA14 was grown in a chemically defined medium (CDM) (70, 71) supplemented with 10 mM KNO_3_ (to support anaerobic respiration), 1 mM Ca, 0.3 µM Mn, 5 µM Fe, 6 µM Zn, 0.1 µM Ni, and 0.1 µM Cu (hereafter termed “metal-replete CDM”). These metal concentrations fall within the reported metal concentrations in CF sputum (72–74). Aerobic cultures were grown in plastic culture tubes without further adjustments. To generate anaerobic conditions, metal-replete CDM inoculated with PA14 in sealed glass serum vials was purged with 5% CO_2_-balanced N_2_ to displace dissolved oxygen from the medium. Aerobic and anaerobic CDM cultures of PA14 were then grown for 8 h and used for gene expression analysis.

We first ensured the purged cultures in serum jars were growing anaerobically by measuring expression of genes known to be either repressed or induced during anaerobic growth. As expected based on previous studies (48, 75), real-time PCR (RT-PCR) showed that transcripts for the pyoverdine sigma factor PvdS and the protease LasA were significantly reduced by strain PA14 in anaerobic cultures compared to aerobic cultures (Fig. S1A). Conversely, transcripts known to be induced during anaerobiosis (69, 75–78)—including transcripts encoding coproporphyrinogen(III) oxidase for anaerobic heme biosynthesis HemN, arginine deaminase ArcA, nitrate reductase precursor NapE, nitrite reductase precursor NirS, and the azurin precursor Azu—were significantly increased in anaerobic cultures (Fig. S1B), demonstrating this system allowed for anaerobic growth of *P. aeruginosa*.

**Fig 1:**
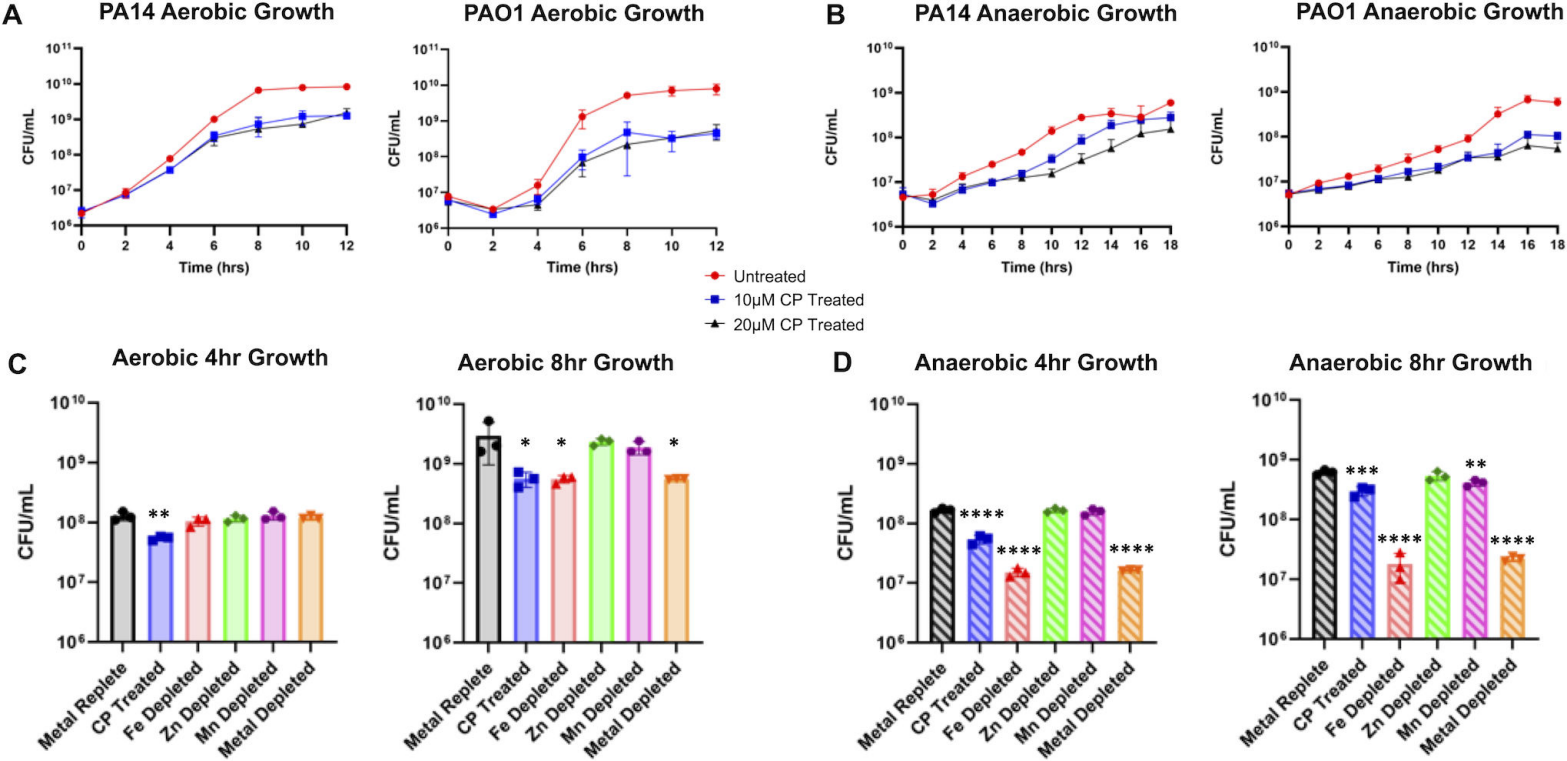
Anaerobic CP treatment of *P. aeruginosa* results in a similar growth defect compared to aerobic conditions. (**A**) Aerobic and (**B**) anaerobic growth curves of strains PA14 and PAO1 grown in metal-replete CDM treated with 0, 10, or 20 µM CP (*n* = 3). Growth of cultures grown in metal replete, 10 µM CP-treated, Fe-depleted, Zn-depleted, Mn-depleted, and metal-depleted CDM under (**C**) aerobic and (**D**) anaerobic conditions. Significance was determined by one-way analysis of variance with Tukey’s multiple comparison test (*n* = 3): *, *P* < 0.05; **, *P* < 0.01; ***, *P* < 0.001; ****, *P* < 0.0001.

We next evaluated the antimicrobial activity of CP against *P. aeruginosa* strains PA14 and PAO1 under anaerobic and aerobic conditions by treating metal-replete CDM cultures with either 0, 10, or 20 µM CP, concentrations that have been reported in biological and clinical samples including CF patient sputum (79). Cultures were sampled every 2 h for enumeration of viable cells. Anaerobic cultures were sampled with a Hamilton gastight syringe to prevent oxygen leakage into the serum vials. As previously observed under aerobic conditions (51), either 10 or 20 µM CP treatment resulted in a reduction of growth for both strains PA14 and PAO1 (Fig. 1A). CP treatment also led to a reduction of growth of both strains under anaerobic conditions (Fig. 1B).

We then tested whether the CP-induced growth defects were due to the sequestration of one or multiple metals. We performed growth assays on cultures of PA14 grown aerobically and anaerobically in the following conditions: metal-replete CDM with or without 10 µM CP; or CDM depleted for iron, zinc, manganese, or all three metals (“metal-depleted”). Viability of aerobic cultures was measured at 4 and 8 h in aerobic conditions and at 10 and 18 h in anaerobic cultures. In aerobic cultures, CP treatment resulted in a small but significant reduction in growth at both 4 and 8 h growth (Fig. 1C). Consistent with previous work from our group (51), aerobic cultures grown in iron- or metal-depleted conditions showed a similar reduction in growth as the CP-treated conditions (Fig. 1C). In contrast, anaerobic cultures grown in iron- or metal-depleted cultures grew more poorly than the CP-treated cultures, with cell culture density doubling only twice by the 18 h time point (Fig. 1D). This observation likely reflects the high requirement of iron for anaerobic nitrate dissimilation and respiration, with iron-depleted cultures surviving via fermentation of arginine present in the growth medium (80, 81). In contrast to our results in aerobic conditions (Fig. 1C), depletion of manganese resulted in a small but significant decrease in anaerobic growth of PA14, suggesting this metal plays a role in anaerobic metabolism (Fig. 1D). Combined, these results demonstrate the requirement for iron as well as the importance of manganese during anaerobic growth of *P. aeruginosa*. Moreover, CP treatment reduces anaerobic growth of *P. aeruginosa* less than iron depletion, possibly due to the ability of cells to efficiently compete with this protein for metals.

### Anaerobic CP treatment results in an iron starvation response that is distinct from the response in aerobic growth

**Fig 2:**
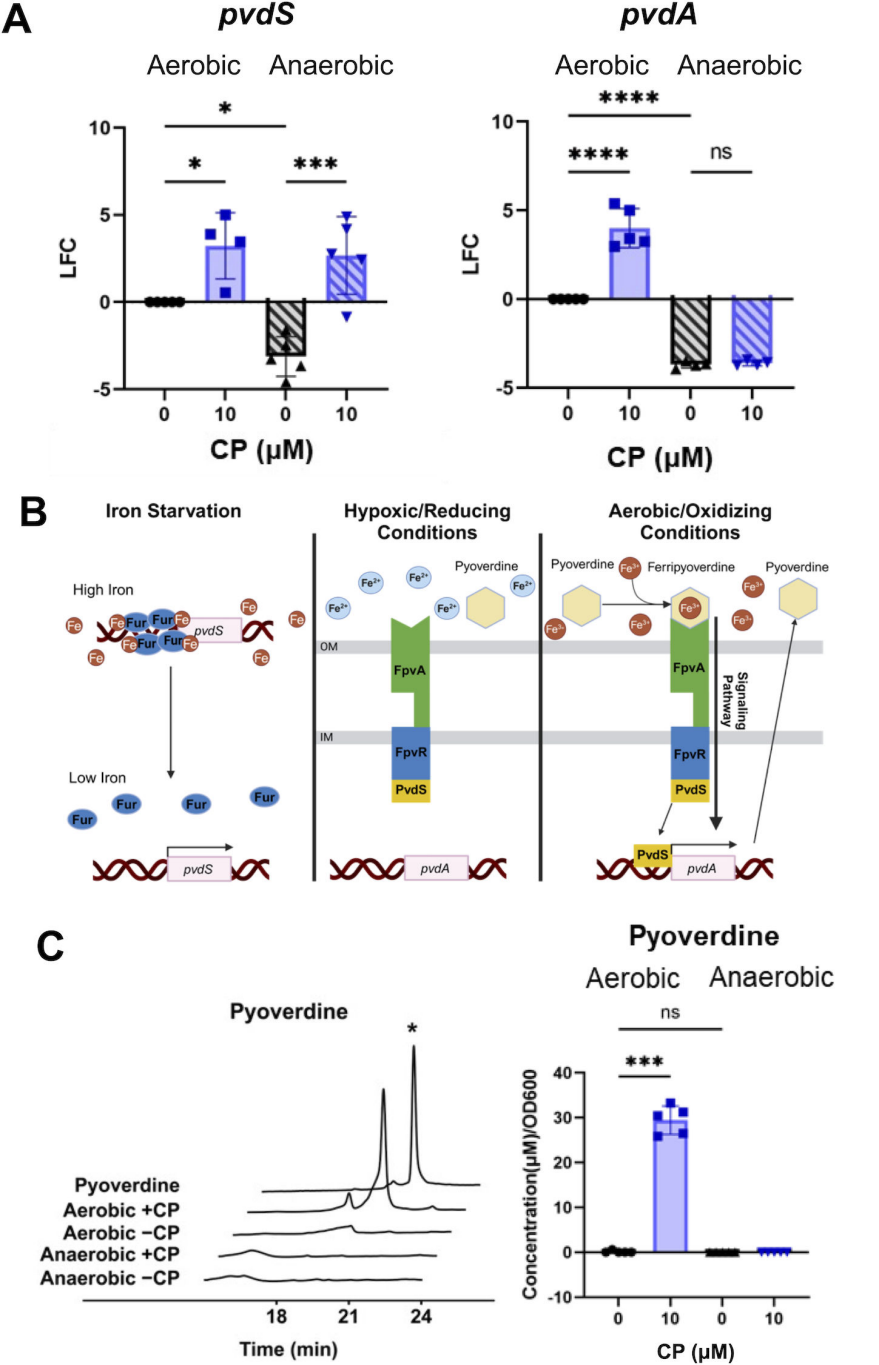
Anaerobic CP treatment leads to induction of *pvdS* expression but not pyoverdine production. (**A**) Gene expression of cultures grown in metal-replete CDM in the presence or absence of 10 µM CP was measured for *pvdS* (left) and *pvdA* (middle) via RT-PCR, and pyoverdine levels were quantified from HPLC of culture supernatant (right). For RT-PCR experiments, the LFC was determined in reference to untreated aerobic cultures. (**B**) Fur regulation of *pvdS* (left), *pvdA* regulation under hypoxic/reducing conditions (middle), *pvdA* regulation under aerobic/oxidizing conditions (right). (**C**) Pyoverdine chromatograms (left) and quantificaiton (right) obtained from HPLC of supernatants from cultures grown under the same conditions (* indicates pyoverdine peak). Significance was determined by one-way analysis of variance with Tukey’s multiple comparison test (*n* = 5): *, *P* < 0.05; **, *P* < 0.01; ***, *P* < 0.001; ****, *P* < 0.0001.

We next evaluated the ability of anaerobic CP treatment to induce iron starvation responses that were previously observed under aerobic conditions (51, 82). We first evaluated the impact of CP on expression of *pvdS*, which, upon its induction, leads to increased production of pyoverdine (83). We expected the effect of CP on the expression these genes would differ for anaerobic conditions, as *pvdS* expression is repressed by the anaerobic regulator Anr (84) and therefore is reduced in anaerobic versus aerobic conditions (Fig. S1A). CP treatment, nevertheless, increased *pvdS* transcript levels by ~6 log_2_ fold change (LFC) (Fig. 2A). PvdS induces expression of *pvdA*, the first gene in the pyoverdine biosynthetic pathway (Fig. 2B) (85, 86). Thus, we next tested whether CP treatment also caused an increase in *pvdA* transcripts. While CP treatment resulted in increased *pvdA* transcript abundance in aerobic conditions, *pvdA* levels were unchanged by CP treatment in anaerobic conditions (Fig. 2A). In agreement with these observations, high-performance liquid chromatography (HPLC) analysis of culture supernatants revealed a prominent peak corresponding to pyoverdine in the aerobic culture supernatants and no detectable peak for pyoverdine in the anaerobic culture supernatants (Fig. 2C). Furthermore, we were only able to quantify pyoverdine in aerobic cultures treated with CP (Fig. 2C). These results are consistent with previous work showing PvdS is only released from its anti-sigma factor FpvR upon uptake of Fe(III)-bound pyoverdine through FpvA (Fig. 2B) (85). Thus, even though *pvdS* is induced by CP treatment anaerobically, pyoverdine is not produced as part of an iron starvation response to CP treatment under anaerobic conditions.

**Fig 3:**
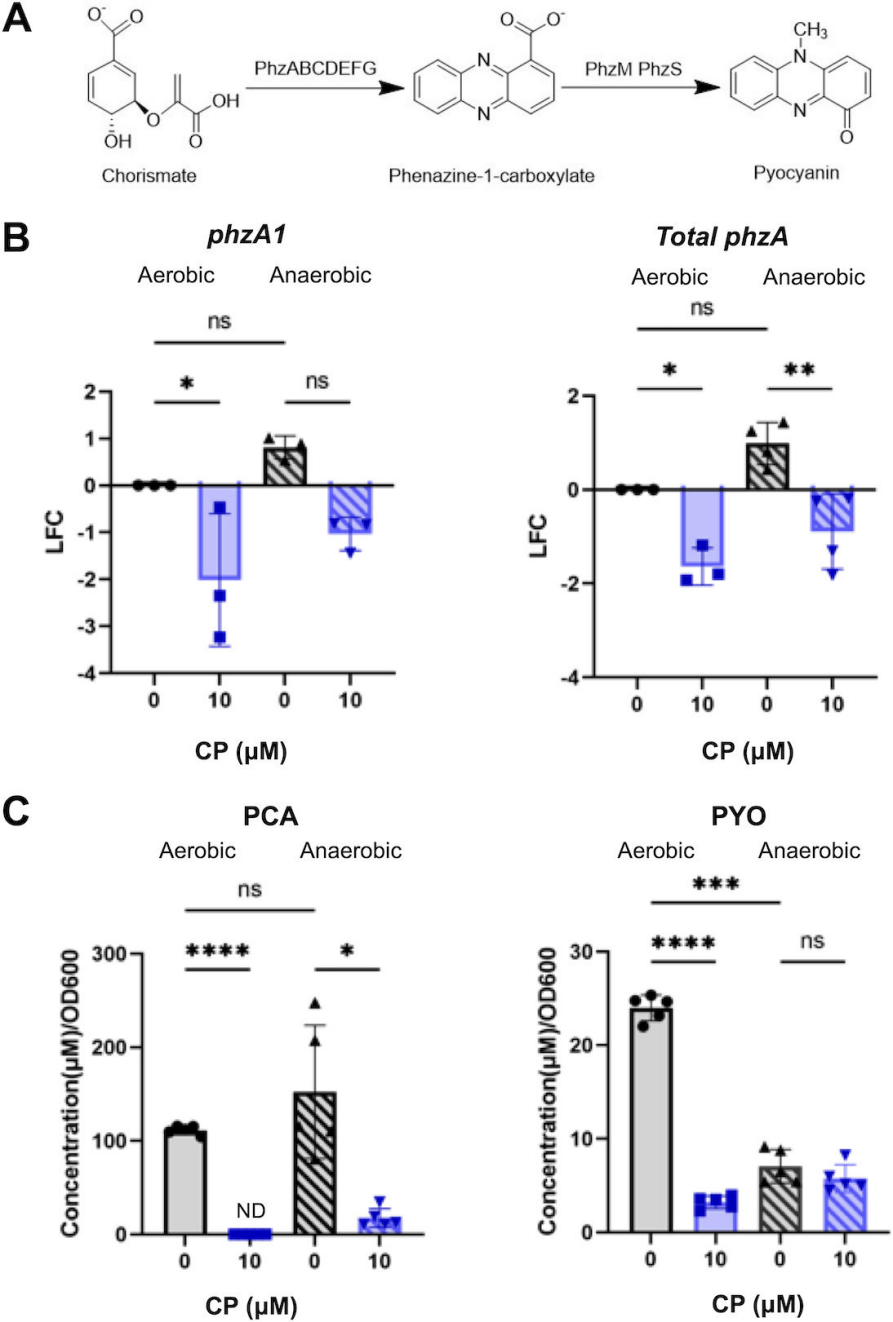
Anaerobic CP treatment represses phenazine production. (**A**) Pathway diagram of the biosynthesis of PCA and PYO. (**B**) Gene expression of cultures grown in metal-replete CDM in the presence or absence of 10 µM CP was measured for *phzA1* (left) and a combination of both *phzA1* and *phzA2* (*phzA* total, right). LFC was determined in reference to untreated aerobic cultures. Metabolite levels of cultures grown under the same conditions were measured via HPLC for (**C**) PCA and (**D**) PYO. Significance was determined by one-way analysis of variance with Tukey’s multiple comparison test (*n* = 3–5): *, *P* < 0.05; **, *P* < 0.01; ***, *P* < 0.001; ****, *P* < 0.0001.

Previous work showed that CP treatment reduces phenazine production by *P. aeruginosa* under aerobic conditions (53, 82), and this effect has been attributed to iron starvation (51, 53, 82). Phenazines are redox cycling secondary metabolites that, among other activities, modulate redox equilibrium and reduce the ratio of Fe(III) to Fe(II), thereby allowing uptake of Fe(II) by the Feo uptake system (33). *P. aeruginosa* produces multiple phenazines from the precursor metabolite chorismate by the biosynthetic enzymes PhzA-G, PhzM, and PhzS (Fig. 3A) (87). The *P. aeruginosa* chromosome contains a duplication in its phenazine biosynthetic genetic cluster, resulting in the *phzA1-G1* (*phz1*) and *phzA2-G2* (*phz2*) operons that are nearly identical (87). Two previously developed sets of primers and probes were developed by our group to analyze expression of the *phz* genes: one set that binds to *phzA1* mRNA specifically and another set that binds to a conserved region of *phzA1* and *phzA2* (88). Using these primer sets, we assessed expression of the *phz* operons by RT-PCR. The resulting data show that CP treatment reduces expression of both *phz* biosynthetic operons under both aerobic and anaerobic conditions, indicating a similar reduction in phenazine production upon CP treatment during anaerobic growth (Fig. 3B). To directly test this idea, we quantified the levels of two phenazines, phenazine-1-carboxylic acid (PCA) and pyocyanin (PYO) (Fig. 3A), by performing analytical HPLC on culture supernatants. Under both aerobic and anaerobic conditions, PCA was produced at high levels by PA14 cultured in metal-replete CDM, and CP treatment similarly reduced PCA levels in both conditions (Fig. 3C). Anaerobiosis resulted in a marked decrease in PYO production (Fig. 3C), which may be explained by the oxygen requirement of the monooxygenase PhzS involved in the conversion of PCA to PYO (Fig. 3A) (87). Consequently, CP treatment had a negligible effect on PYO levels in anaerobic cultures supernatants. We attribute decreased PCA production to a non-canonical iron starvation response as has been previously reported (51).

**Fig 4:**
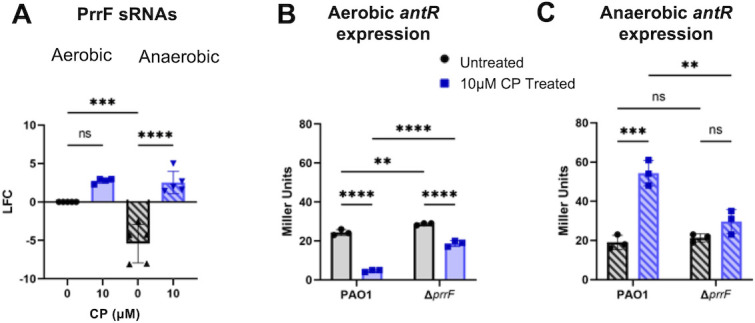
Anaerobic CP treatment results in changes to the PrrF-mediated iron-sparing response. (**A**) Expression of the PrrF sRNAs in cultures grown in metal-replete CDM in the absence or presence of 10 µM CP measured via RT-PCR. LFC was determined in reference to untreated aerobic cultures. β-Galactosidase activity of *P. aeruginosa* PAO1/P*_antR_*-UTR*_antR_*-‘*lacZ* and Δ*prrF*1,2/P*_antR_*-UTR*_antR_*-‘*lacZ* grown in metal-replete CDM in the absence or presence of 10 µM CP under (**B**) aerobic and (**C**) anaerobic conditions. Significance was determined by one-way analysis of variance with Tukey’s multiple comparison test (*n* = 3–5): *, *P* < 0.05; **, *P* < 0.01; ***, *P* < 0.001; ****, *P* < 0.0001.

We next determined if CP treatment during anaerobic growth led to induction of the PrrF-mediated iron-sparing response (39, 51, 88). RT-PCR analysis revealed significant induction of the iron-repressed PrrF sRNAs (8 LFC) upon anaerobic CP treatment (Fig. 4A). Prior studies demonstrated that PrrF blocks the expression of genes encoding iron-containing proteins by binding to and reducing stability of their target mRNAs, a process dubbed the iron-sparing response (39, 88). To ascertain if CP treatment resulted in a similar iron-sparing response anaerobically, we analyzed expression of *antR*, which is known to be directly inhibited by the PrrF sRNAs (89). To measure *antR* expression, we used a translational reporter strain containing the promoter and 5´ UTR sequence of *antR* fused to the *lacZ* coding region in both WT PAO1 (PAO1/P*_antR_*-UTR*_antR_*-‘*lacZ*) and PAO1Δ*prrF* (Δ*prrF*1,2/P*_antR_*-UTR*_antR_*-‘*lacZ*) strains (89). Using the same conditions as above, we found that CP treatment led to PrrF-dependent reduction in *antR* reporter activity under aerobic conditions, as shown previously (Fig. 4B) (51, 89). By contrast, CP treatment of cultures grown anaerobically resulted in a significant increase in *antR* reporter activity (Fig. 4C). This induction of *antR* by CP under anaerobic conditions was lost in the Δ*prrF1,2* strain, suggesting the increase is PrrF dependent (Fig. 4C). Since the PrrF sRNAs are produced both aerobically and anaerobically, and it is known that PrrF downregulates *antR* expression aerobically, these data suggest that there are differences in PrrF regulation under aerobic and anaerobic conditions.

We next aimed to compare the iron starvation responses elicited by CP to those that occur upon iron depletion, as we have done previously under aerobic conditions (53, 54). For this analysis, we examined gene expression with varied concentrations of iron in the culture medium. Surprisingly, iron supplementation (0.05 µM–5.0 µM iron) had no significant effect on either *pvdS* or *prrF* expression levels (Fig. S2A). We next examined whether CP sequestration of metals other than iron might be responsible for the iron starvation responses observed upon CP treatment. In this experiment, we observed a small (0.5 LFC) induction of the PrrF sRNAs upon iron starvation (Fig. S2B), much lower than the ~5 LFC observed with CP treatment (Fig. 4A). We speculate that anaerobic metabolism by *P. aeruginosa* requires higher levels of iron, and that depletion of iron below our metal-replete concentration of 5 µM leads to less healthy growth and erratic gene expression changes. By contrast, iron may still be acquired in the presence of CP, allowing for healthier growth and a more effective iron starvation response.

To test the ability of CP-treated cultures to acquire iron, we performed inductively-coupled plasma mass spectrometry (ICP-MS) on harvested cells from aerobic and anaerobic metal-replete CDM cultures treated with either 10 or 20 µM CP for 8 h. Consistent with previous studies (51), treatment of aerobic cultures with either 10 or 20 µM CP reduced cell-associated iron concentrations, while concentrations of manganese, zinc, and copper showed no significant change (Fig. S3A and B). Notably, anaerobic CP treatment resulted in no significant decrease of any cell-associated metals, including iron (Fig. S3C and D). Thus, it appears that *P. aeruginosa* maintains its iron quota in CP-treated anaerobic cultures. Cell-associated levels of zinc were variable between experiments, preventing any conclusion regarding the effect of CP on zinc uptake. We also ascertained the metal inventories of individual and multi-metal-depleted cultures (Fig. S4). As expected, we observed significant reductions in cell-associated iron and zinc concentrations in the iron- and zinc-depleted cultures, respectively, and reductions in the cell-associated levels of both metals in the metal-depleted cultures (Fig. S4A and B).

The anaerobic growth system for this study uses glass serum vials; since glass can carry metal contamination, we repeated the experiment using plastic serum vials and found that cell-associated iron levels in anaerobic cultures grown in either plastic or glass vials were similar (Fig. S3E and F, 2–6 µM/optical density at 600 nm [OD_600_]). We also performed anaerobic growth assays on PA14 cultured in either glass or plastic serum vials, revealing enhanced growth that resembled growth in aerobic conditions (Fig. S5A and B compared to Fig. 1C and D). These data suggest that oxygen permeated the plastic vials, making the cultures less sensitive to iron depletion than under strict anaerobiosis. Based on these results, we proceeded with glass serum vials for the remainder of the study.

In summary, these data demonstrate the impact of CP on anaerobic metal homeostasis is distinct from what occurs during aerobic growth. The anaerobic response to CP included a lack of PVD production under anaerobic CP treatment, and an altered iron-sparing response by the PrrF sRNAs. These data also demonstrate the *P. aeruginosa*’s strict requirement for iron during anaerobic growth, indicating different metabolic requirements in the absence of oxygen.

### Transcriptomic response of PA14 to anaerobic CP treatment reveals changes in iron uptake strategies

**Fig 5:**
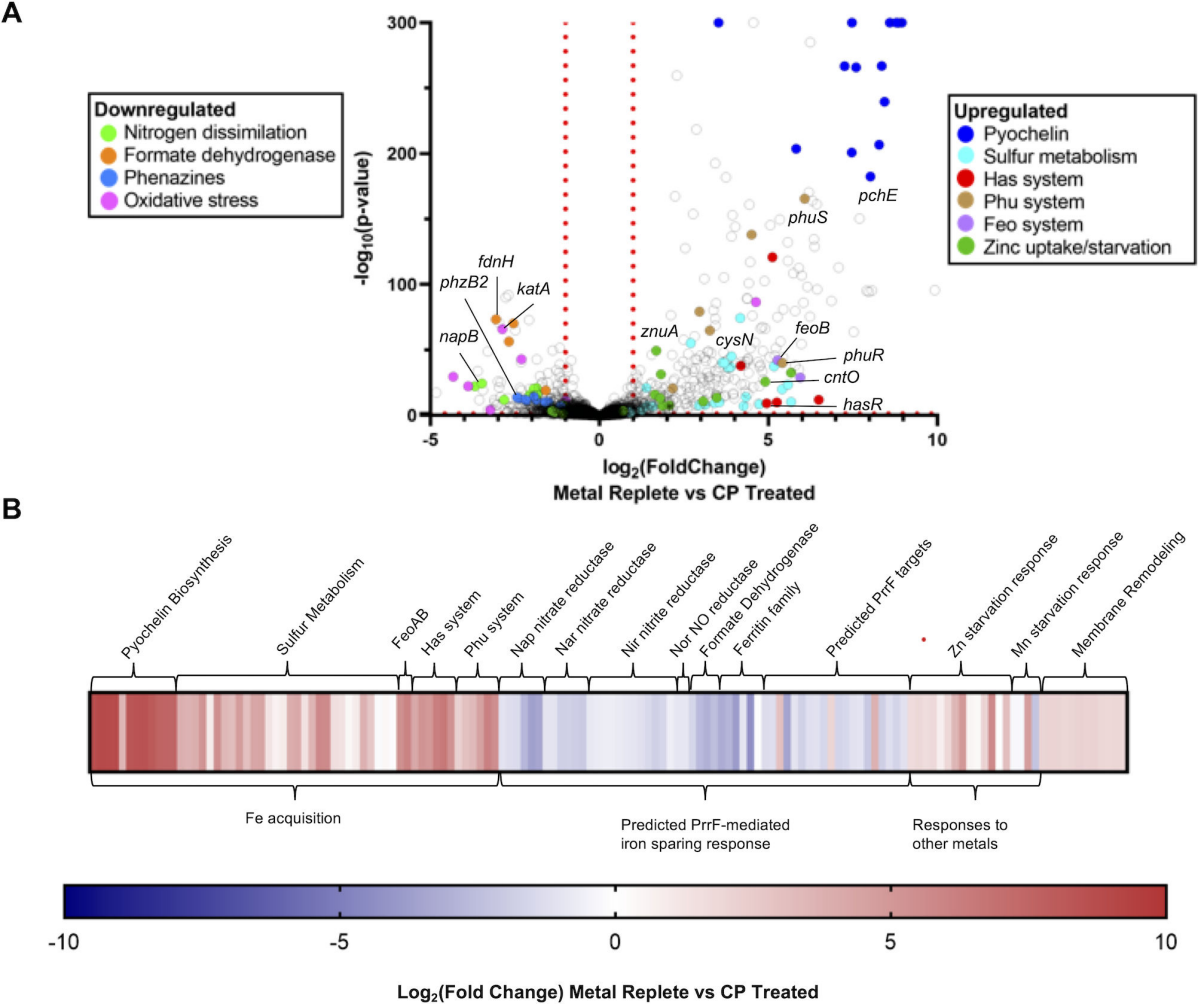
Transcriptomic analysis of *P. aeruginosa* response to anaerobic CP treatment. (**A**) Volcano plot of PA14 response to CP treatment. (**B**) Heat map displaying gene expression of systems of interest.

To further understand how CP affects overall metal homeostasis and *P. aeruginosa* physiology in anaerobic conditions, we evaluated the impact of 10 µM CP on the PA14 transcriptome during anaerobic growth. PA14 was cultured anaerobically in glass serum vials with metal-replete CDM in the presence or absence of 10 µM CP. Cells were harvested after 8 h and total RNA was extracted for RNAseq using Illumina HiSeq (2 × 150 bp reads) as previously described (45, 90). Transcripts that were significantly changed by CP treatment by at least 1 LFC were analyzed further as described below. Using this cut-off, CP treatment significantly induced 376 genes and repressed 460 genes. The observed changes included hallmarks of a robust iron starvation response, including the induction of genes for siderophore synthesis and heme uptake and the downregulation of genes encoding iron-containing proteins (Fig. 5). However, we noted several differences in this response compared to prior aerobic CP treatment and iron starvation studies (53, 88). Specific systems affected by CP treatment are presented with further analysis below.

#### Induction of pyochelin biosynthetic genes

**Fig 6:**
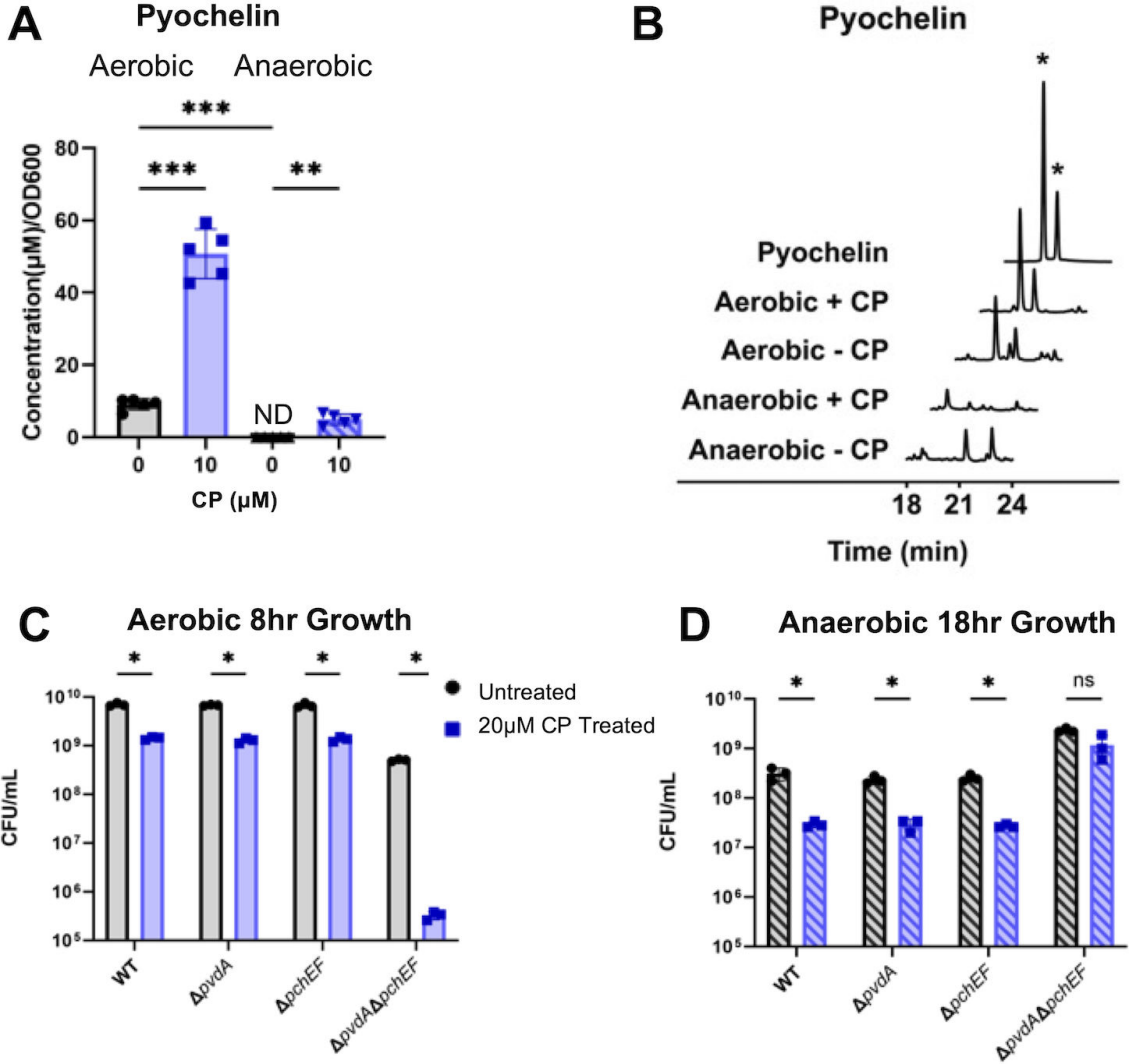
Siderophore biosynthetic genes are associated with a fitness cost during anaerobic growth. Pyochelin levels of cultures grown in metal-replete CDM treated with 0 or 10 µM CP were quantified (**A**) from the chromatograms obtained from HPLC of culture supernatants (**B**) where the two peaks marked by * are diastereomers of pyochelin (96). Growth of wild-type PAO1, Δ*pvdA*, Δ*pchEF*, and Δ*pvdA*Δ*pchEF* grown aerobically (**C**) and anaerobically (**D**) in metal-replete CDM in the presence or absence of 20 µM CP. For HPLC quantification, significance was determined by one-way analysis of variance (ANOVA) with Tukey’s multiple comparison test (*n* = 5). For growth experiments, significance was determined by two-way ANOVA with Tukey’s multiple comparison test (*n* = 3): *, *P* < 0.05; **, *P* < 0.01; ***, *P* < 0.001; ****, *P* < 0.0001.

CP treatment resulted in a robust induction of genes for pyochelin biosynthesis under anaerobic conditions, as well as genes for sulfur metabolism (Fig. 5A and B). Previous proteomic studies of the aerobic iron starvation response showed no induction of pyochelin genes upon CP treatment or iron starvation (53, 88). Moreover, both CP treatment and iron depletion resulted in a downregulation of the sulfur metabolism pathway, consistent with previous studies (53, 88). Sulfur metabolism is linked to pyochelin biosynthesis through the conversion of sulfate, taurine, and alkanesulfonate into L-cysteine (Fig. S6A) (91, 92). The pyochelin non-ribosomal peptide synthetase then converts L-cysteine to pyochelin (Fig. S6A) (93). To further evaluate the induction of pyochelin and sulfur metabolism genes during anaerobic growth, we monitored expression of genes for pyochelin biosynthesis (*pchE*) and sulfur metabolism (*cysN*) in both anaerobic and aerobic conditions after 8 h of growth. In agreement with the RNAseq results, CP treatment upregulated *pchE* expression in anaerobic but not aerobic cultures grown for 8 h (Fig. S6B and C). Next, to determine if pyochelin was produced in response to CP treatment in aerobic and anaerobic PA14 cultures, we performed HPLC analysis of the culture supernatants. Aerobic culture supernatants showed a significant increase in pyochelin levels in response to CP treatment, whereas CP treatment of anaerobic cultures resulted in very low levels of pyochelin (Fig. 6A and B). Based on earlier studies showing that pyochelin is produced upon moderate iron starvation and pyoverdine upon more severe iron starvation (31, 94, 95), we hypothesized that CP induction of pyochelin synthesis genes in aerobic cultures may have occurred at time points prior to when we harvested the cultures. We performed a time-course study and found that, in agreement with this notion, maximal *pchE* expression was reached in CP-treated cultures after 4 h of aerobic growth and then gradually decreased over time (Fig. S6C). This trend was consistent for *cysN*, with maximal expression upon CP treatment also observed at 4 h of aerobic growth (Fig. S6D). Therefore, we concluded that under aerobic conditions, CP induction of pyochelin biosynthesis occurs during exponential phase rather than during stationary phase, while the timing of induction of pyochelin gene expression in anaerobic growth is delayed to later time points.

Since siderophore production is substantially reduced in anaerobically CP-treated cultures, we tested whether these systems affect anaerobic growth of *P. aeruginosa* upon CP treatment. We performed growth assays on *P. aeruginosa* wild-type strain PAO1 and isogenic deletion mutants lacking the ability to produce either pyoverdine (∆*pvdA*) or pyochelin (∆*pchEF),* as well as a double mutant that does not produce either siderophore (∆*pvdA*∆*pchEF*) (97). Aerobic and anaerobic cultures were grown in metal-replete CDM in the presence or absence of 20 µM CP, and viability was determined by enumerating colony-forming units (CFU) after 10 and 18 h of growth. Consistent with our previous growth analysis (Fig. 1B), CP treatment reduced growth of wild-type PAO1 in both aerobic and anaerobic conditions (Fig. 6C and D). CP treatment similarly reduced growth of strains lacking genes for the biosynthesis of individual siderophores (Fig. 6C and D). The loss of the ability to produce both siderophores led to a more significant growth defect during aerobic growth, even in the absence of CP treatment (Fig. 6C). Unexpectedly, under anaerobic conditions, the ∆*pvdA*∆*pchEF* strain grew to higher levels than the wild-type or single mutant strains, both in the presence and absence of CP (Fig. 6D). This induction of growth indicates that production of siderophores is detrimental to *P. aeruginosa* growth under anaerobic conditions.

#### Feo-mediated iron uptake

**Fig 7:**
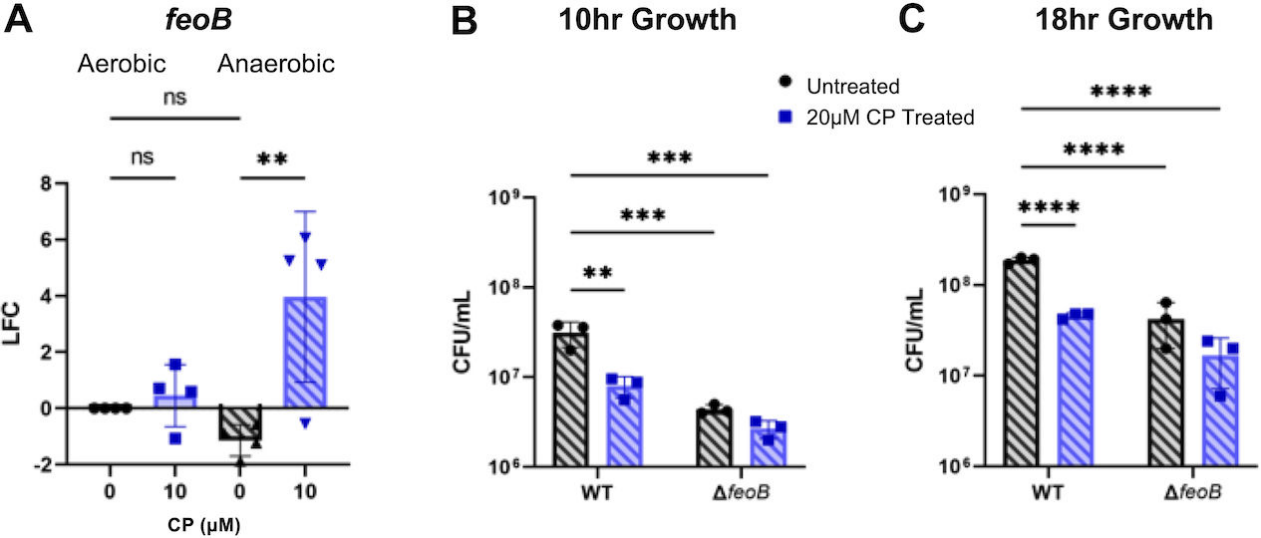
The FeoAB system is required for optimal anaerobic growth. (**A**) *FeoB* expression of cultures grown in metal-replete CDM in the presence or absence of 10 µM CP measured via RT-PCR. LFC was determined in reference to untreated aerobic cultures. Significance was determined by one-way analysis of variance (ANOVA) with Tukey’s multiple comparison test (*n* = 4): *, *P* < 0.05; **, *P* < 0.01; ***, *P* < 0.001; ****, *P* < 0.0001. Growth of wild-type PAO1 and Δ*feoB* grown anaerobically in metal-replete CDM in the presence or absence of 20 µM CP after (**B**) 10 h and (**C**) 18 h. Significance was determined by two-way ANOVA with Tukey’s multiple comparison test (*n* = 3): *, *P* < 0.05; **, *P* < 0.01; ***, *P* < 0.001; ****, *P* < 0.0001.

RNAseq revealed that anaerobic CP treatment strongly upregulated genes encoding the inorganic Fe(II) uptake system (Feo) (Fig. 5A and B). Subsequent RT-PCR analysis demonstrated that this upregulation is specific to anaerobic conditions, as shown previously (Fig. 7A) (32, 33, 98–100). To evaluate the impact of Feo on anaerobic growth, we performed growth assays of a PAO1 deletion mutant lacking the *feoB* gene (∆*feoB*) in the absence or presence of CP as described above. CP treatment resulted in reduced cell density in wildtype cultures grown for either 10 or 18 h (Fig. 7B and C). The Δ*feoB* mutant showed overall reduced anaerobic culture density compared to wild type in both untreated and CP-treated cultures (Fig. 7B and C). While density of the ∆*feoB* culture was slightly reduced in the presence of CP, this decrease was not statistically significant (Fig. 7B and C). These results show that regardless of the presence of CP, the Feo system is important for optimal growth under anaerobic conditions.

#### Heme acquisition

**Fig 8:**
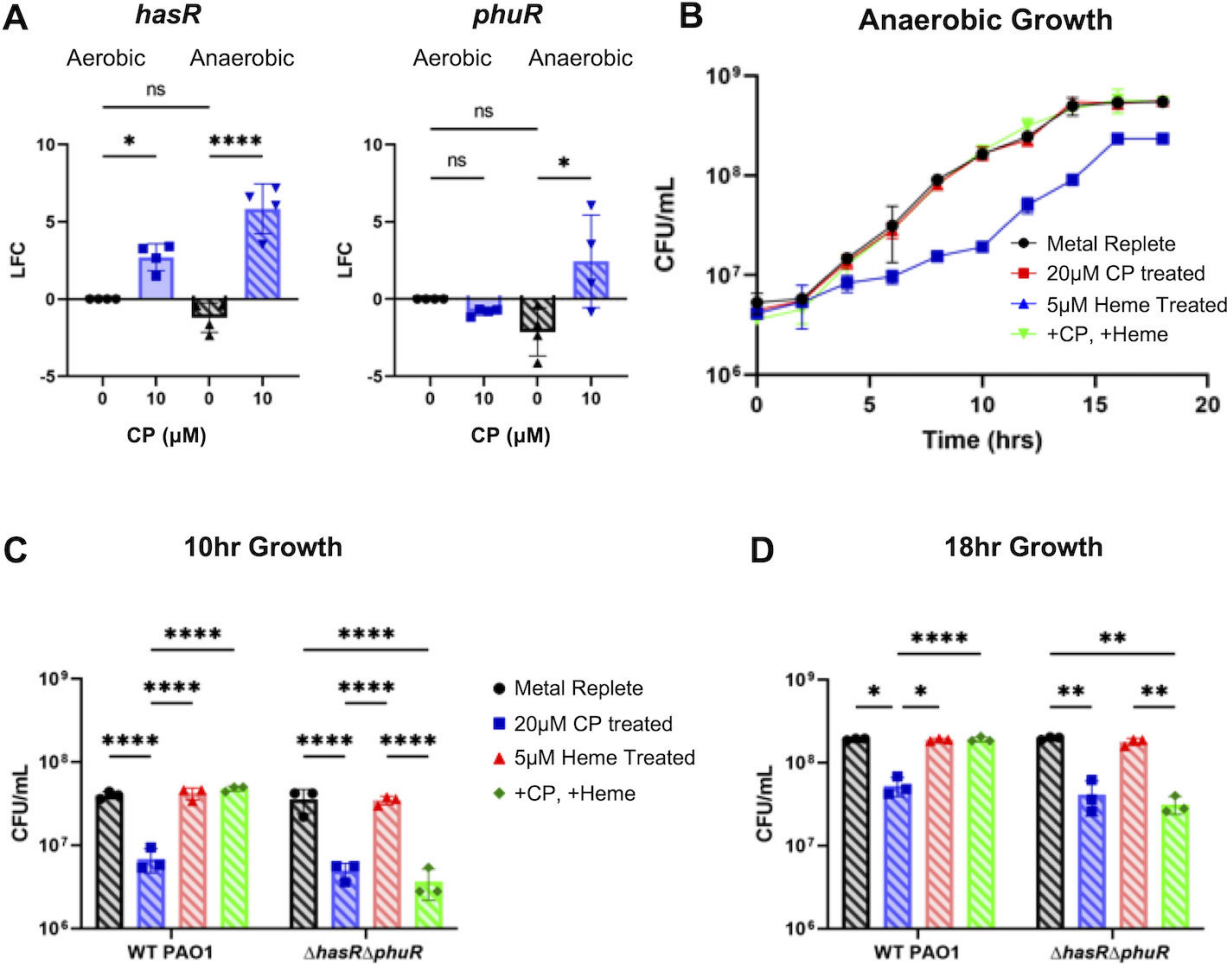
Anaerobic CP treatment induces *hasR* and *phuR* gene expression, which rescues *P. aeruginosa* from CP-induced growth defects in the presence of heme. (**A**) Gene expression of *hasR* (left) and *phuR* (right) in cultures grown in metal-replete CDM in the presence or absence of 10 µM CP measured via RT-PCR. LFC was determined in reference to untreated aerobic cultures. Significance was determined by one-way analysis of variance (ANOVA) with Tukey’s multiple comparison test (*n* = 4): *, *P* < 0.05, **, *P* < 0.01, ***, *P* < 0.001, ****, *P* < 0.0001. (**B**) Anaerobic growth curve of PA14 grown in metal-replete CDM and metal-replete CDM treated with either 20 µM CP, 5 µM heme, or a combination of 20 µM CP and 5 µM heme. (**C and D**) Anaerobic growth of wild-type PAO1, and PAO1 Δ*hasR*Δ*phuR* grown under the same conditions after 10 h (**C**) and 18 h (**D**) of growth. Significance was determined by two-way ANOVA with Tukey’s multiple comparison test (*n* = 3): *, *P* < 0.05, **, *P* < 0.01, ***, *P* < 0.001, ****, *P* < 0.0001.

Genes for both the Has and Phu systems were strongly induced by anaerobic CP treatment (Fig. 5A and B). These genes included *hasAp* encoding a secreted hemophore, *hasR* encoding the hemophore receptor, and *phuR* encoding a heme transporter (34, 35). Previous studies have shown that while induction of the Has system is highly sensitive to iron starvation in aerobic conditions, expression of *phuR* is dependent on the presence of heme (34, 35). RT-PCR analysis demonstrated that under aerobic conditions, *hasR*, but not *phuR*, is induced by CP treatment, whereas anaerobic CP treatment resulted in robust upregulation of both genes, despite the absence of heme (Fig. 8A). These data suggest that *P. aeruginosa* may rely more on heme uptake during anaerobic growth, particularly in the presence of CP.

To evaluate this idea, we performed growth assays to determine if heme protects *P. aeruginosa* from iron withholding by CP, as was observed in previous aerobic studies (54). We grew cultures of PA14 anaerobically in untreated metal-replete CDM with or without supplementation of 20 µM CP, 5 µM heme, or both CP and heme, and we enumerated CFU at 2 h intervals over 18 h of growth. Anaerobic cultures treated with 20 µM CP showed the expected reduction in growth (Fig. 8B). The addition of 5 µM heme did not provide any additional growth benefits compared to the untreated metal-replete cultures (Fig. 8B). However, and in agreement with previous aerobic studies (54), heme supplementation restored growth of CP-treated cultures, indicating that the presence of heme also protects *P. aeruginosa* from the antimicrobial activity of CP in anaerobic environments (Fig. 8B).

To determine the impact of the Has and Phu systems on anaerobic growth, we performed growth assays on PAO1 deletion mutants for either the HasR transporter (∆*hasR*), the PhuR transporter (∆*phuR*), or a double mutant lacking both transporters (∆*hasR*∆*phuR*). Mutants were grown under the same conditions as above. After 10 and 18 h, the wild-type strain, single ∆*hasR* and ∆*phuR* mutants, and double ∆*hasR*∆*phuR* mutant cultures showed comparable reductions in cell density upon CP treatment (Fig. 8C and D; Fig. S7). The effect of CP on wild-type and single heme uptake mutants was rescued by heme supplementation (Fig. S7). By contrast, heme supplementation had no effect on culture density of the double ∆*hasR*∆*phuR* culture treated with CP (Fig. 8C and D). These data demonstrate that while the individual heme uptake systems can compensate for one another in anaerobic conditions, the loss of both systems prevents *P. aeruginosa* from using heme as an alternative iron source to overcome CP-induced antimicrobial activity.

#### PrrF-mediated iron-sparing responses

Anaerobic CP treatment led to a decrease in transcripts encoding systems that were previously computationally shown to share complementarity with the PrrF sRNAs via CopraRNA (88). These included the *nap* genes encoding nitrate reductase, *nir* genes encoding nitrite reductase, and *fdn* genes encoding nitrate-inducible formate dehydrogenase (Fig. 5A and B). Anaerobic CP treatment resulted in identification of additional genes that were downregulated by CP treatment and shared computationally predicted PrrF complementarity determined by CopraRNA analysis. These genes included *tagJ1* from the Hcp secretion island (HSI)-I type VI secretion system (T6SS), *icmF2* from the HSI-II T6SS, and *tadZ* encoding a type IVb pilin assembly protein (Fig. S8). We tested whether CP-induced downregulation of these genes is dependent on the PrrF sRNAs by performing RT-PCR analysis on anaerobic cultures of PAO1 and the isogenic Δ*prrF*, but our results showed no discernible loss of this regulation in the ∆*prrF* mutant (Fig. S8).

#### PrrH-mediated heme homeostasis

**Fig 9:**
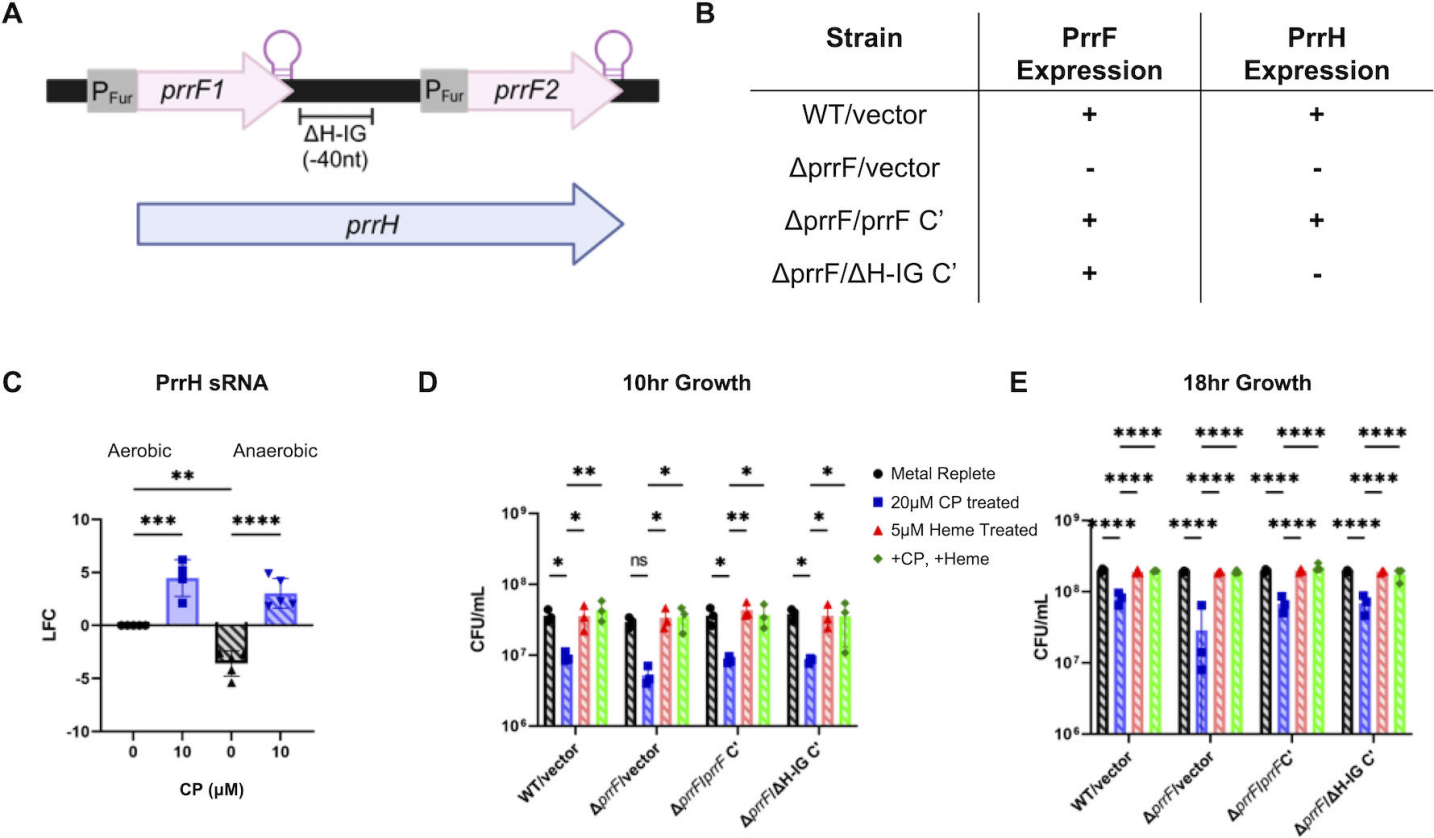
The ability to produce the PrrF sRNAs but not PrrH is needed for growth to be fully restored by heme. (**A**) Diagram of the *prrF* operon. (**B**) Table of strains used, displaying their ability to express both PrrF and PrrH. (**C**) Gene expression of PrrH in cultures grown in metal-replete CDM in the presence or absence of 10 µM CP measured via RT-PCR. LFC was determined in reference to untreated aerobic cultures. Significance was determined by one-way analysis of variance (ANOVA) with Tukey’s multiple comparison test (*n* = 5): *, *P* < 0.05; **, *P* < 0.01; ***, *P* < 0.001; ****, *P* < 0.0001. Anaerobic growth curve of WT/vector, Δ*prrF*/vector, Δ*prrF*/*prrF* C’, and Δ*prrF*/ΔH-IG C’ grown in metal-replete CDM and metal-replete CDM treated with either 20 µM CP, 5 µM heme, or a combination of 20 µM CP and 5 µM heme after (**D**) 10 h and (**E**) 18 h. Significance was determined by two-way ANOVA with Tukey’s multiple comparison test (*n* = 3): *, *P* < 0.05; **, *P* < 0.01; ***, *P* < 0.001; ****, *P* < 0.0001.

The genes for the PrrF1 and PrrF2 sRNAs are located in tandem, and read-through transcription of the *prrF1* terminator yields a third heme-responsive sRNA, PrrH (Fig. 9A). PrrH is upregulated when heme is present (46), and recent data suggest it downregulates pyochelin biosynthesis genes (45). The *prrH* sequence is highly conserved among all sequenced *P. aeruginosa* isolates (97). As heme becomes an increasingly important iron source during CF lung infection (98, 101, 102), it is hypothesized that heme regulation via PrrH may be important in conditions relevant to chronic infection (45). Therefore, we measured the expression of PrrH in aerobic and anaerobic PA14 cultures grown in metal-replete CDM in the absence or presence of 10 µM CP. We used previously designed RT-PCR primers and probes that bind and amplify the sequence intergenic to *prrF1* and *prrF2*; thus they are specific for the PrrH sRNA (Fig. 9A) (45). We also performed growth assays on mutant strains of PAO1 that differ in their ability to produce the PrrF and PrrH sRNAs (Fig. 9B). When comparing the untreated cultures, anaerobic growth led to overall lower levels of PrrH expression, though CP treatment still led to a robust induction of the PrrH sRNA in both aerobic and anaerobic conditions (Fig. 9C).

We then tested whether mutation of the PrrH sRNA would affect growth in anaerobic CP-treated cultures using a *prrF* locus allele that lacks 40 nt of the *prrF1-prrF2* intergenic region, termed *prrF*∆H-IG (Fig. 9A) (45). This allele was then used to complement the ∆*prrF* mutant, alongside complementation with a wild-type *prrF* allele (Fig. 9B). Previous work shows that both complementation constructs restore PrrF expression and rescue ∆*prrF* phenotypes, while the ∆H-IG allele produces a truncated PrrH sRNA (45). The complemented ∆*prrF* mutant strains, as well as wild-type PAO1 and ∆*prrF* both carrying the pUCP18 vector, were grown anaerobically as described above. After both 10 and 18 h of growth, all strains showed decreased growth when treated with CP (Fig. 9D and E). This phenotype was more pronounced in the ∆*prrF*/vector control strain at 18 h, and complementation with either the wild-type or ∆H-IG alleles restored growth to wild-type levels (Fig. 9E). Heme restored growth of all CP-treated cultures (Fig. 9D and E). These data indicate that the PrrF sRNAs, but not PrrH, play a role in survival of *P. aeruginosa* during anaerobic CP treatment, but only in the absence of heme.

#### Zinc and manganese starvation responses

RNAseq revealed that CP also elicited a zinc starvation response under anaerobic conditions, though this response was modest compared to the iron starvation response (Fig. 5A and B). This response included the upregulation of genes encoding alkaline proteases and zinc uptake and homeostasis genes (Fig. 5A and B). To further evaluate the impact of CP on zinc starvation responses in anaerobic versus aerobic conditions, RT-PCR was performed on genes involved in zinc uptake. We analyzed the expression of *znuA* encoding a member of the zinc uptake system (103), and we found that while aerobic CP treatment resulted in significantly increased *znuA* expression, no induction was observed by CP treatment under anaerobic conditions (Fig. S9A). Another method of zinc uptake is through the metallophore pseudopaline, biosynthesis of which is encoded by the *cntIMLO* operon (104). In contrast to aerobic CP treatment, anaerobic CP treatment resulted in no significant induction of the *cntO* gene encoding the pseudopaline transporter (Fig. S9B). These results suggest that CP induces a more modest Zn starvation response in anaerobic conditions, possibly due to a decreased need for this metal as compared to aerobic growth.

**Fig 10:**
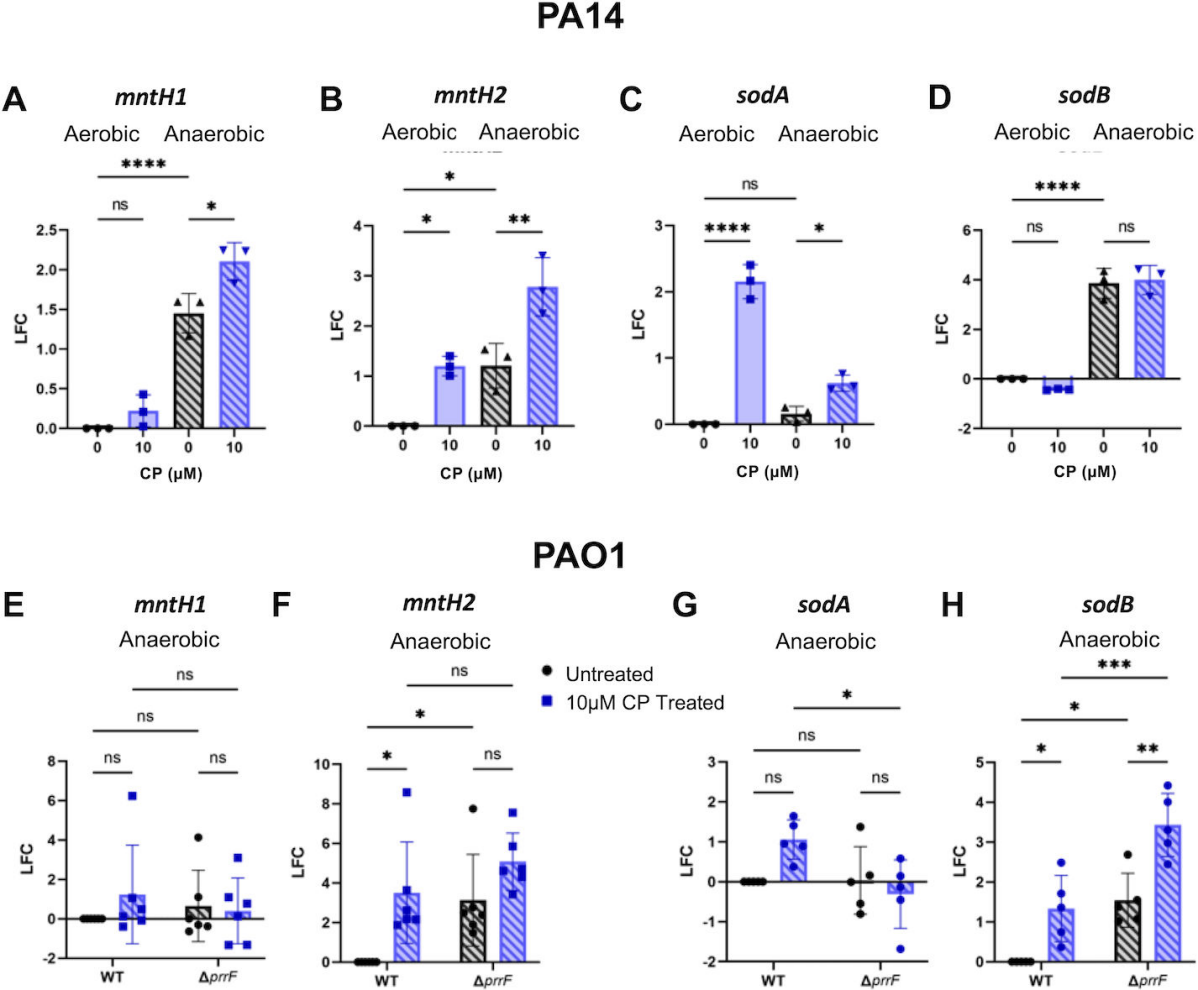
CP treatment induces a manganese starvation response under anaerobic conditions. Gene expression of (**A, E**) *mntH1,* (**B, F**) *mntH2,* (**C,G**) *sodA*, and (**D, H**) *sodB in* cultures of PA14, PAO1, and PAO1 ∆*prrF* grown aerobically and anaerobically as indicated in metal-replete CDM in the absence or presence of 10 µM CP measured via RT-PCR. LFC was determined in reference to untreated aerobic cultures. Significance was determined by one-way analysis of variance with Tukey’s multiple comparison test (*n* = 3): *, *P* < 0.05; **, *P* < 0.01; ***, *P* < 0.001; ****, *P* < 0.0001.

While RNAseq did not show any evidence of a manganese starvation response, the perhaps higher sensitivity of RT-PCR revealed a significant induction of both predicted manganese transporters *mntH1* and *mntH2* (105) upon anaerobic CP treatment, and an overall increase in both mRNAs in anaerobic versus aerobic conditions (Fig. 10A and B). To determine if there were other indications of manganese starvation upon anaerobic CP treatment, we also analyzed the expression of *sodA* encoding the manganese-cofactored superoxide dismutase and *sodB* encoding the iron-cofactored superoxide dismutase. SodA has been previously shown to be upregulated under low iron conditions to compensate for the reduced expression of the iron-cofactored superoxide dismutase SodB (88, 106). In agreement with this finding, CP treatment resulted in increased *sodA* expression in both aerobic and anaerobic conditions, though the induction was not as robust in anaerobic cultures (Fig. 10C). Moreover, anaerobic growth resulted in lower overall transcript levels of *sodA* and higher overall transcript levels of *sodB* (Fig. 10C and D). The finding that anaerobic CP treatment results in a robust induction of the genes for both predicted manganese transporters, combined with reduced overall *sodA* expression and increased overall *sodB* expression during anaerobic growth, suggests an increased requirement for manganese during anaerobic growth.

We previously discovered that the PrrF sRNAs mediate regulatory cross-talk between iron and zinc. Specifically, the PrrF sRNAs are required for iron induction of gene PA5535, encoding a putative P-loop GTPase that is also induced by zinc starvation, and we identified complementarity between PrrF and the 5´ end of the PA5535 mRNA (88). We therefore wondered if PrrF might similarly affect the putative manganese starvation response elicited by anaerobic CP treatment. To test this idea, we grew anaerobic cultures of PAO1 and PAO1Δ*prrF* in metal-replete CDM in the presence or absence of CP. In contrast to what was observed in PA14, neither anaerobiosis nor CP treatment resulted in increased *mntH1* expression in PAO1 (Fig. 10E). However, anaerobic CP treatment led to a significant upregulation of *mntH2* in PAO1, and deletion of *prrF* led to de-repression of *mntH2* in the absence of CP treatment (Fig. 10F). We did not identify any complementarity between PrrF and *mntH2*, but these data indicate a novel PrrF regulatory effect on the expression of potential manganese transporters.

Lastly, we questioned whether PrrF was responsible for CP-dependent induction of the gene for the manganese-cofactored SodA under anaerobic conditions. While we noted a ~1 LFC induction of *sodA* upon CP treatment in PAO1, this increase was not statistically significant (Fig. 10G). However, we did observe a significant decrease in *sodA* expression in the Δ*prrF* mutant compared to wild type (Fig. 10G), again suggesting a role for PrrF in regulating potential manganese starvation genes. We also tested the impact of *prrF* deletion on CP-dependent induction of *sodB*, a known target of the PrrF sRNAs. In contrast to what was observed in PA14 (Fig. 10D), expression of *sodB* was strongly induced upon CP treatment during anaerobic growth (Fig. 10H). While overall *sodB* levels were increased in the Δ*prrF* mutant compared to the wild type, we still noted a significant upregulation of *sodB* in the ∆*prrF* mutant treated with CP, indicating the additional presence of PrrF-independent regulatory pathways affecting anaerobic *sodB* expression. Combined, these data indicate that the PrrF sRNAs play a role in what appears to be a manganese starvation response upon anaerobic CP treatment, though further studies are required to understand the underlying mechanisms for these responses.

#### Putative multi-metal responses

RNAseq revealed that CP treatment also induced expression of genes encoding membrane remodeling proteins (Fig. 5B). These genes include the *pmrAB* two-component system which modulates resistance to cationic antimicrobial peptides, as well as the spermidine biosynthesis genes *speD2* and *speE2* involved in the addition of the polyamine spermidine to lipid A and the *arn* operon involved in the addition of aminoarabinose to lipid A (Fig. S10A and B) (107, 108). The upregulation of membrane remodeling genes is consistent with our previous aerobic analysis showing these genes are upregulated in response to CP (53). In the previous study, we found that induction of membrane remodeling genes is not linked to depletion of a single metal but instead appeared to be due to depletion of multiple metals (53). We were unable to determine if the same is true under anaerobic conditions due to our inability to remove iron from the media and obtain robust expression data. Nonetheless, the consistency of this response as well as previous studies (109, 110) suggest that CP exerts a similar effect on membrane modification systems during aerogic and anaerobic growth.

#### Summary of transcriptomics

**Fig 11:**
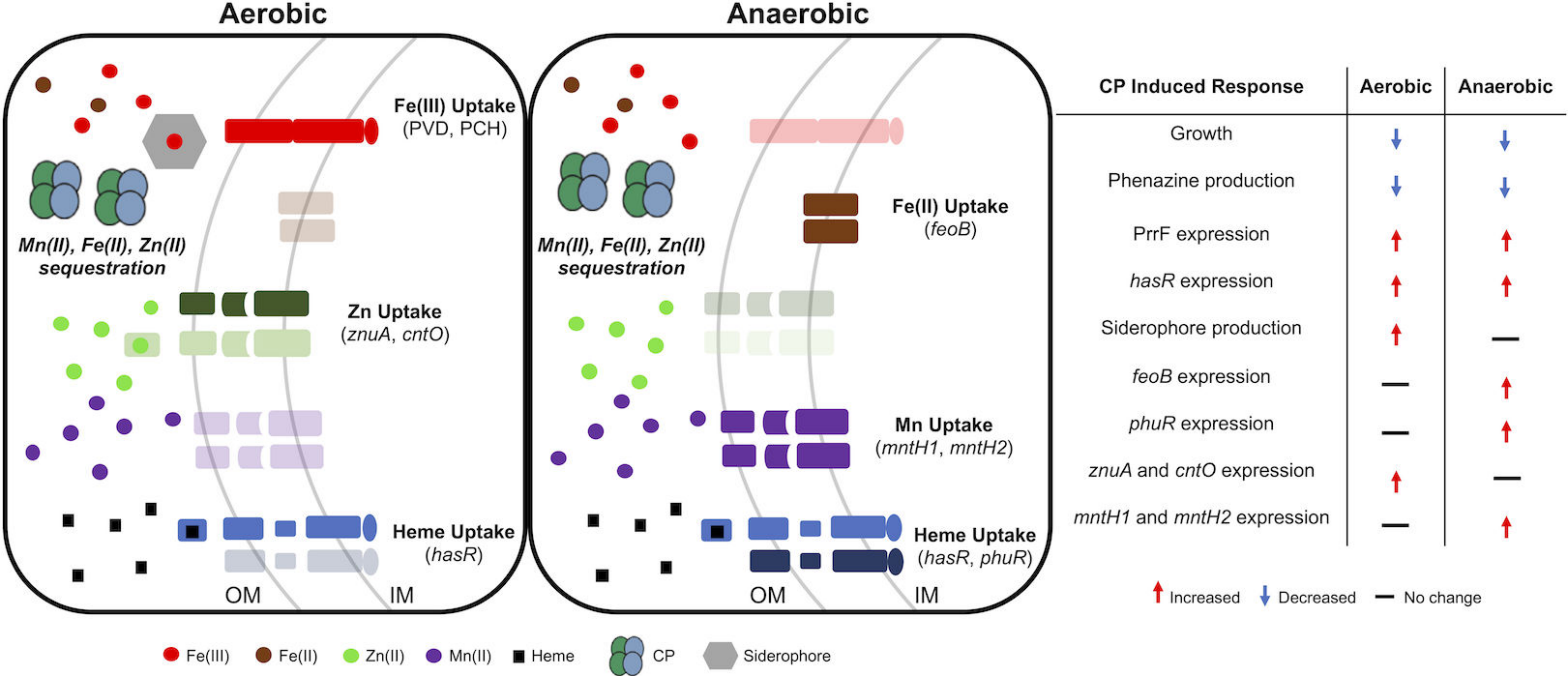
Model of *P. aeruginosa* responses to CP in aerobic and anaerobic environments. Table shows which CP-induced responses are increased, decreased, or unchanged in aerobic and anaerobic conditions.

Altogether, the transcriptomic and subsequent analyses revealed that CP treatment has wide-ranging effects on PA14 gene expression under both aerobic and anaerobic conditions (Fig. 11). While most of these responses indicate anaerobic CP treatment results in a robust iron starvation response in *P. aeruginosa*, we also observed modest responses indicative of Zn and Mn withholding. Our results also identify *mntH2* and *sodA* as potential targets of the PrrF sRNAs in anaerobic conditions and outline a potential role for PrrF in the manganese starvation response during anaerobic growth. These results provide compelling evidence that the absence of oxygen alters the *P. aeruginosa’s* response to CP exposure, potentially due to altered metal requirements.

## DISCUSSION

The current study demonstrates how the absence of oxygen changes *P. aeruginosa’s* response to metal withholding by CP, and provides further insight into how its metal homeostasis pathways adapt to hypoxic environments. This work identifies several distinctions of the anaerobic response of *P. aeruginosa* to CP, including upregulation of the FeoAB Fe(II) and PhuR heme uptake systems, substantially reduced siderophore production, and altered PrrF regulatory impacts during anaerobic growth. Moreover, anaerobic CP treatment induces what appears to be a stronger manganese starvation response and less robust zinc starvation response than what is observed aerobically. Overall, this study demonstrates that anaerobiosis substantially alters metal homeostasis in *P. aeruginosa*.

*P. aeruginosa* acquires iron by different strategies that depend on the surrounding environment and the iron sources available (101). Under aerobic or oxidizing conditions, the prevalent source of iron is Fe(III), for which siderophores can compete with host immune proteins such as lactoferrin (24, 31). Siderophores have long been a target for antimicrobial development (18, 19), yet our current study indicates that siderophore production may prove costly to microbial growth in anaerobic conditions that often dominate advanced CF lung infection. This finding is consistent with studies showing the differential importance of siderophores versus heme and Fe(II) update in acute infections compared to chronic CF lung infections (98, 111). Indeed, previous studies demonstrate that genes encoding the Feo and Phu systems are consistently upregulated in *P. aeruginosa* isolated from CF infections (32, 97, 98, 102).

These differences have implications on the design of novel antimicrobials that either rely on iron sequestration or targeting specific iron uptake systems (20). One consideration is the use of therapies that sequester Fe(III), such as deferoxamine, or that target siderophore uptake, such as antibiotic siderophore conjugates (19–22). These therapies may function differently during acute and chronic infection where the latter has reduced Fe(III) concentrations. By contrast, strategies that target heme uptake, including recently reported gallium-salophen heme mimics (23), may prove more beneficial during chronic CF lung infection where heme is a more prevalent source of iron (97, 102). Furthermore, oxygen gradients within biofilm communities likely alter the iron sources available throughout the environment, highlighting the possible utility of a multi-pronged approach to disrupting iron uptake. Thus, understanding the complexities of iron uptake systems as well as the environmental context of different pseudomonal infections is critical during drug development and design.

*P. aeruginosa* mediates iron homeostasis through various strategies. When cytosolic iron levels are high, the Fur protein downregulates expression of iron uptake systems and increases expression of oxidative stress protection pathways; when iron is limiting, expression of iron uptake systems is derepressed, and the PrrF-mediated iron-sparing response is activated (37, 39, 46, 85). In this study, we observed differences in the PrrF-mediated iron-sparing response between aerobic and anaerobic conditions, particularly with regard to *antR* reporter activity. Thus, we propose that PrrF may function differently in anaerobic conditions, which is reasonable as metabolic demands that drive iron requirements are substantially altered during anaerobiosis. Notably, we found that induction of *mntH2* was dependent on PrrF, suggesting regulatory cross-talk between iron and manganese, similar to what we found during aerobic conditions between iron and zinc (88). Defining how oxygen availability affects PrrF sRNA function will help elucidate the distinct response of *P. aeruginosa* to metal starvation during anaerobic growth.

By using defined medium conditions, we found that iron depletion nearly eliminated growth of *P. aeruginosa* during anaerobiosis, highlighting the high iron requirement for anaerobic respiration. *P. aeruginosa* cannot grow anaerobically in the absence of nitrate as a terminal electron acceptor and instead survives via arginine fermentation (81, 112). By contrast, many facultatively aerobic pathogens, including those in the Enterobacteriaceae, ferment carbohydrates to support anaerobic growth. Previous work revealed increased Fur repression in anaerobic *Escherichia coli* K12 cultures, correlated with increased labile Fe(II) in the cell (113, 114). However, these studies did not evaluate the impact of iron being absent from the medium nor the impact of fermentation on these results, raising questions of whether fermentative growth in anaerobic conditions has an absolute iron requirement. Strict and facultative anaerobes, including the fermentative pathogen *Clostridioides difficile*, rely on the function of ferrosomes to store iron and maintain iron homeostasis during anaerobic growth (55, 115–117). The presence of CP increases the expression of genes responsible for ferrosome assembly *in vitro* and *in vivo* (55), though additional studies are required to elucidate how *C. difficile* ferrosomes contribute to iron homeostasis in anaerobic conditions.

Our data also suggest distinct requirements for zinc and manganese during anaerobic growth as compared to aerobic growth. While the induction of zinc starvation responses was muted in anaerobic compared to aerobic growth, these results are reminiscent of a previous study showing a reduction of zinc uptake in *E. coli* grown anaerobically (118). By contrast, anaerobic CP treatment resulted in the upregulation of *mntH1* and *mntH2*, encoding predicted manganese transporters (119). Currently, little is known about *P. aeruginosa* manganese homeostasis. Remarkably, one study reported that CP lacks antimicrobial activity against *Salmonella* when grown anaerobically, a result that was attributed to the inessential role of the manganese-cofactored SodA when oxygen was absent (67). While our results indicate reduced expression of *sodA* in anaerobic *P. aeruginosa* cultures, our data also suggest an increased reliance on manganese during anaerobiosis. Metabolic requirements for anaerobic growth of *Salmonella* and *P. aeruginosa* are distinct (fermentation versus respiration, respectively), which may explain the difference in CP antimicrobial activity in these studies.

Similar to previous studies (53), anaerobic CP treatment led to gene expression changes associated with membrane remodeling. The membrane remodeling response involves upregulation of the PmrAB system, which in response to low magnesium levels induces the expression of lipid A modification systems (107, 120). These systems include the Arn operon, which is involved in the addition of aminoarabinose to lipid A, and Spe genes that are involved in the addition of polyamine spermidine to lipid A, processes associated with *P. aeruginosa* immune evasion and resistance to antimicrobial peptides (107, 120–122). Under aerobic conditions, induction of these genes upon CP treatment was concluded to be due to depletion of multiple metals; however, simultaneous depletion of manganese, zinc, and iron in this previous study did not elicit the same level of induction as did CP treatment, suggesting this induction could also be due to a metal-independent mechanism. At present, we are unable to ascertain if this response under anaerobic growth conditions is metal dependent or independent. Previous studies showed that upregulation of the Arn operon results in aminoarabinose-modified, positively-charged lipopolysaccharide (LPS), which confers resistance to colistin (121), while increased spermidine biosynthesis stabilizes the outer membrane, conferring resistance to antibiotics and oxidative damage induced by the host immune system (122). Since these responses persist in low oxygen environments, it is likely they will remain important in hypoxic and anoxic conditions present at chronic infection sites. Together, these results suggest that *P. aeruginosa* recognizes CP as a host immune protein and responds by inducing these immune evasion systems.

Overall, this study provides critical insight into how *P. aeruginosa* manages metal homeostasis in response to CP treatment during anaerobiosis, a critical yet understudied set of environmental conditions relevant to CF lung infections. This work also demonstrates the challenges of studying metal homeostasis under anaerobic conditions where metal requirements appear to be altered, necessitating more investigations into how *P. aeruginosa* adapts its metal requirements to oxygen depletion. Notably, while many of the responses uncovered in our transcriptomics study can be attributed to CP withholding of specific metals, the stimulus for other responses remains unknown. We further expect these studies to enhance studies into how *P. aeruginosa* responds to CP in biofilm communities, which exhibit a steep oxygen gradient (123, 124). Thus, this study broadens our understanding of *P. aeruginosa* metal homeostasis, anaerobic lifestyle, and potential host-pathogen interactions that may be more relevant to chronic infections.

## MATERIALS AND METHODS

### Bacterial strains, media, and conditions

Strains used in this study are listed in Table S1. *P. aeruginosa* was grown in a previously described CDM (51, 70, 71). CDM was supplemented with 10 mM KNO_3_, 1 mM CaCl_2_, 0.1 µM CuCl_2_, 0.1 µM NiCl_2_, 5 µM FeSO_4_, 6 µM ZnCl_2_, and 0.3 µM MnCl_2_ to afford metal-replete CDM. CP was added at 10 or 20 µM as indicated. Fe-depleted, Zn-depleted, and Mn-depleted CDM was made by omitting Fe, Zn, or Mn, respectively, from the metal-replete CDM. Metal-depleted CDM was made by omitting Fe, Zn, and Mn from the media. *P. aeruginosa* lab strains of PAO1, PA14, and their deletion mutants were routinely grown overnight by streaking from freezer stocks in tryptic soy agar or brain heart infusion (BHI) agar (Sigma, St. Louis, MO) plates and incubated at 37°C for 12 h–16 h. Three to five colonies were taken from each streaked plate and inoculated in 2 mL of metal-replete CDM and incubated for 12 h–16 h with shaking at 250 rpm at 37°C. Aerobic cultures were grown in 1.5 mL of metal-replete CDM in 15 mL plastic culture tubes as previously described (53). Anaerobic cultures were grown by optimizing a previously described protocol (68, 69). Anaerobic cultures were grown in 5 mL CDM prepared in acid-washed 30 mL glass serum vials. The vials were capped with rubber stoppers and the cultures purged with 5% CO_2_-balanced N_2_ for 2 h to remove oxygen from both the media and head space. Cultures for growth assays were inoculated to an OD_600_ of 0.005 and grown for 18 h with shaking at 250 rpm at 37°C. Cultures for all other assays were inoculated to an OD_600_ of 0.05 and grown for 8 h with shaking at 250 rpm at 37°C. All experiments were performed with at least three biological replicates to ensure reproducibility.

### Calprotectin purification

CP was purified as previously described (59). CP aliquots were stored in 20 mM HEPES, 100 mM NaCl, and 5 mM dithiothreitol (pH 8.0) at −80°C. Aliquots were thawed only before use and buffer exchanged three times with modified Tris buffer (MTB) (20 mM Tris, 100 mM NaCl, pH 8.0) using sterile 10 kDa molecular weight cut-off spin concentrators (Pierce). CP concentrations were determined by A_280_ using the extinction coefficient of the CP heterodimer (ε_280_ = 18,450 M^−1^cm^−1^) obtained from the online ExPASy ProtParam tool.

### Heme stock preparation

For assays with heme supplementation, a fresh stock of hemin (Sigma) was prepared within 30 min of the assay and concentration was determined using a modified pyridine hemochrome assay (125). A small amount of hemin was washed with 1 mL of 100 mM HCl twice to remove free iron and then dissolved in 200 µL of 200 mM NaOH. The dissolved hemin was then diluted in Tris buffer (pH 7.4–8) and sterilized with a 0.2 µm syringe filter. To measure the concentration the prepared heme, a 1:1,000 dilution was prepared with 899 µL of sterile MilliQ water, 1 µL of heme, 100 µL pyridine (Sigma), and a small amount of sodium dithionate (Sigma), and the absorbance at 418, 525, and 555 nm was measured. The heme concentration was determined using calculated extinction coefficients for pyridine hemochrome (170 mM^−1^cm^−1^ at 418 nm, 17.5 mM^−1^cm^−1^ at 425 nm, and 34.5 mM^−1^cm^−1^).

### Metal inventory assay

Five cultures of *P. aeruginosa* strain PA14 were grown anaerobically as described above in metal-replete CDM supplemented with either 10 or 20 µM CP. Untreated cultures were supplemented with an equal amount of MTB. Cultures were incubated for 8 h with shaking at 250 rpm at 37°C before measuring the OD_600_ of the cultures and harvesting cells by centrifugation (4,000 rpm, 4°C, 7 min). Cells were washed three times with 1 mL cold (i) MTB, (ii) MTB + 500 µM EDTA, and (iii) MTB buffer. Cells were resuspended in 2 mL 5% HNO_3_ and stored at 4°C. The acidified samples were liquified by microwave digestion and analyzed by ICP-MS. Reported metal concentrations are normalized to the culture’s OD_600_. Both individual data points and the mean metal content are reported with *P*-values from a two-tailed *t*-test assuming unequal variances.

### ICP-MS

Metal concentrations were quantified with an Agilent 7900 inductively coupled plasma-mass spectrometer located in the Center for Environmental Health Sciences Bioanalytical Core facility at the Massachusetts Institute of Technology. The instrument was operated in helium mode. The instrument was calibrated before each analysis using five serially diluted (1:10) samples of the environmental calibration standard (Agilent) in 5% nitric acid (Honeywell, TraceSELECT; >69.0%) as well as a 5% nitric acid-only standard. The concentrations of Fe, Zn, Mn, Ni, and Cu were quantified and terbium (1 ppb terbium; Agilent) was used as an internal standard. Samples were prepared in 5 mL Eppendorf tubes, transferred to ICP-MS polypropylene vials (PerkinElmer Life Sciences), and analyzed.

### CP and heme supplementation growth assays

Three cultures of *P. aeruginosa* strain PA14 were grown anaerobically as previously described in metal-replete CDM. Cultures were supplemented with 10 µM CP, 20 µM CP, 5 µM heme, or a combination of 20 µM CP and 5 µM heme as indicated. Growth was monitored by taking 50 µL aliquots with Air-Tite syringes (Hamilton) every 2 h for 18 h. Aliquots were immediately serially diluted in sterile phosphate-buffered saline, plated on BHI agar plates, and incubated for 18 h–24 h at 37°C. Isolated colonies on each plate were enumerated and used to calculate the amount of colony forming units in 1 mL of culture (CFU per milliliter).

### Growth assays

Three cultures of *P. aeruginosa* strain PAO1 and deletion mutants were grown aerobically or anaerobically as previously described in metal-replete CDM. Cultures were supplemented with 20 µM CP, 5 µM heme, or a combination of 20 µM CP and 5 µM heme as indicated. Fifty microliter aliquots of each culture were taken at 0, 10, and 18 h. Aliquots were serially diluted, and plated as previously described. CFU per milliliter of each culture was determined as previously described.

### High-performance liquid chromatography

HPLC was performed on an Agilent 1200 instrument equipped with a thermally controlled autosampler compartment (4°C). The solvents used for all chromatography were (A) H_2_O/0.1% trifluoroacetic acid (TFA) and (B) MeCN/0.1% TFA. For analytical HPLC, a CLIPEUS C18 5 µm reverse-phase column (Higgins Analytical, 5µm pore size, 4.6 × 250mm, catalog number CS-2546-C185) was used for separation. A flow rate of 1 mL/min was used. For analysis of pyochelin, pyocyanin, and phenazine-1-carboxylate, the method used was 0–8 min = 0% B, 8–10 min = 15% B, 10–30 min = 15%−80% B, 30–32 min = 80%–100% B, 32–38 min = 100% B, 38–40 min = 100%–0% B, 40–48 min = 0% B, with absorbance detection set at 220 nm, 310 nm, and 365 nm. For analysis of pyoverdine, the method used was 0–8 min = 0% B, 8–38 min = 0%–35% B, 38–42 min = 35%–100% B, 42–46 min = 100% B, 46–50 min = 100%–0% B, 50–58 min = 0% B, with fluorescence detection with excitation at 398 nm and detecting emission at 455 nm.

For semi-preparative HPLC, 300–900 µL aliquots of samples were injected onto a Zorbax 300 SB C18 reverse-phase column (Agilent, 5 µm pore size, 9.4 × 250 mm, 880995-202) for separation. A flow rate of 4 mL/min was used, and absorbance was monitored at 220 nm and 280 nm. For purification of pyoverdine, the method used was 0–2 min = 0% B, 2–2.5 min = 0%–8% B, 2.5–5 min = 8% B, 5–25 min = 8%–13% B, 25–26 min = 13%–95% B, 26–30 min = 95% B, 30–31 min = 95%–0% B, 31–35 min = 0% B. For purification of pyochelin, the method used was 0–8 min = 0% B, 8–10 min = 0%–15% B, 10–30min = 15–80% B, 30–32 min = 80%–100% B, 32–36 min = 100% B, 36–40 min = 100%–0% B, 40–48 min = 0% B.

For each analyzed metabolite, standard curves (for quantification) were prepared by dissolving commercially available or purified compounds isolated from bacterial culture supernatants in 1:1 solvent A:B. Pyocyanin and phenazine-1-carboxylic acid were obtained from Sigma. Pyoverdine was extracted and purified as previously reported (51). Pyochelin was extracted and purified using a modified protocol adapted from previous reports (see below) (51, 96, 126).

### Preparation of culture supernatants for HPLC analysis

Aliquots (200 µl) of culture suspensions were collected in polypropylene Eppendorf tubes and centrifuged at 4,000 rpm, for 7 min, at 4°C to remove the majority of cells/debris. The clarified supernatant was transferred into a new tube and stored at −20°C. Supernatants were thawed on ice and a 125 µL aliquot of supernatant was diluted 1:1 with 125 µL of ice-cold HPLC-grade methanol (Sigma, 34860-1L-R) for a final volume of 250 µL. The diluted samples were stored at −20°C for 1 h to precipitate salt, following which they were centrifuged at 13,000 rpm, for 10 min, at 4°C to pellet any particles. Two hundred microliters of the supernatant was carefully transferred into a new ice-cold tube and centrifuged again at 13,000 rpm, for 10 min, at 4°C to ensure complete removal of any particulates. One hundred fifty microliters of the supernatant was transferred into an HPLC vial fitted with a glass insert, and kept on ice until analysis.

### Isolation of pyochelin

A single colony of *P. aeruginosa* PAO1 (on L agar) was used to inoculate a sterile, 250 mL baffled flask containing 40 mL of Tris/tryptic soy broth medium supplemented with 2 mM Ca(II). 2,2-Bipyridyl (Sigma, D216305) was added from a 200 mM stock in dimethyl sulfoxide (DMSO) for a final working concentration of 600 µM. The culture was incubated for 24 h, at 37°C, at 250 rpm, following which the cells were pelleted by centrifugation at 13,000 rpm, for 10 min, at 4°C. The supernatant was removed and stored at −20°C for 20–24 h. A Sep-Pak C18 Plus Long Cartridge (Waters, WAT023635) was equilibrated with 40 mL of 20% methanol (in solvent A, MilliQ water with 0.1% TFA), following which the cartridge was loaded with 35 mL of supernatant. The cartridge was washed with 10 mL of solvent A, and eluted with (i) 10 mL of 20% methanol and (ii) 10 mL of 40% methanol. Fractions were checked for pyochelin content using liquid-chromatography followed by mass spectrometry (LC-MS), and fractions containing pyochelin were combined. Methanol was removed using a rotary evaporator and the remaining solvent was removed by lyophilization. Pyochelin was purified by a semi-preparative HPLC, eluting as two broad peaks (diastereomers based on the chiral center at the thiazolidine ring) at around 45% B. The semipure material was lyophilized and further purified using analytical HPLC, affording two sharp peaks at around 45% B. To confirm the identity of these species, we analyzed fractions corresponding to each peak by Orbitrap high-resolution mass spectrometry. We found that each analyzed fraction gave two diastereomeric peaks (suggesting some degree of epimerization in solvent) which matched the expected exact mass for pyochelin (Table S3). The two fractions were combined, lyophilized to dryness, dissolved in 1:1 solvent A/B, and stored at −20°C. The concentration of the resulting pyochelin stock was determined by UV-vis absorbance measurements in methanol, detected at 310 nm (ε_310_ = 4,200 M^−1^cm^−1^) (127).

### Real-time PCR

Three to five cultures of PA14 were grown as previously described in metal-replete CDM, Fe-depleted CDM, Zn-depleted CDM, Fe- and Zn-depleted CDM, or metal-replete CDM treated with 10 µM CP with or without the presence of oxygen as indicated. RT-PCR was performed as previously described (40). Briefly, harvested cultures were stored in RNAlater at −80°C. RNA was extracted using the Qiagen RNeasy kit, cDNA was synthesized, and RT-PCR was performed using TaqMan reagents (Roche) and a StepOnePlus system (Thermo Fisher). Standard curves were produced for each primer-probe set listed in Table S2 in the supplemental material by analyzing cDNA generated from serial dilutions of RNA and used to determine relative amounts of RNAs as described previously (40). Relative RNA levels were then normalized to the levels of 16S ribosomal RNA.

### RNAseq

Three cultures of PA14 were grown as described above in metal-replete CDM or metal-replete CDM in the presence of 10 µM CP with or without the presence of oxygen as indicated. RNA samples were prepared as previously described (90). All further sample preparation and analysis were performed by Azenta Life Sciences. RNA integrity was validated using an Agilent 2100 Bioanalyzer, and libraries were prepared with samples having a minimum RNA integrity number of 8. Ribosomal RNA was removed using an Illumina Ribo-Zero kit, and samples were converted into Illumina sequencing libraries using the ScriptSeq v2 RNA-Seq Library Preparation Kit (Epicentre, Illumina). Libraries were sequenced using Illumina HiSeq (2 × 150 bp reads) with an average of 77 million reads for each sample. Sequence reads were trimmed to remove possible adapter sequences and nucleotides with poor quality using Trimmomatic v.0.36. The trimmed reads were then mapped to the reference genome of *P. aeruginosa* strain PA14 (GCA_000014625) available on ENSEMBL using the Bowtie2 aligner v.2.2.6. Unique gene hit counts were calculated by using featureCounts from the Subread package v.1.5.2. Only unique reads that fell within gene regions were counted. The gene hit counts were used for downstream differential expression analysis. Using DESeq2, a comparison of gene expression between CP-treated and untreated samples was performed. The Wald test was used to generate *P*-values and LFC. Genes with an adjusted *P*-value less than 0.05 and an absolute LFC greater than 1 were called as differentially expressed genes for each comparison.

### Network analysis

Network analysis was performed using the STRING database version 12.0 (128). Corresponding PAO1 accession numbers were used as the database works exclusively with the PAO1 strain.

## Data Availability

The RNAseq differential expression data have been deposited to the NIH BioProject database with the accession number PRJNA1151675.

## SUPPLEMENTAL MATERIAL

Supplemental tables and figures jb.00029-25-s0001.pdf Tables S1 to S5; Figures S1 to S8.

Supplemental Dataset jb.00029-25-s0002.xlsx RNAseq data.

ASM does not own the copyrights to Supplemental Material that may be linked to, or accessed through, an article. The authors have granted ASM a non-exclusive, world-wide license to publish the Supplemental Material files. Please contact the corresponding author directly for reuse.
