# Supplementary material for: Calprotectin elicits aberrant iron starvation responses in Pseudomonas aeruginosa under anaerobic conditions
Source: J Bacteriol. 2025 Mar 26;207(4):e00029-25. doi: 10.1128/jb.00029-25 (PMC12004955; doi:10.1128/jb.00029-25)
Supplement: Supplemental tables and figures — Tables S1 to S5; Figures S1 to S8. [file jb.00029-25-s0001.pdf]

Supplementary materials for:

Calprotectin elicits aberrant iron starvation responses in *Pseudomonas aeruginosa* under anaerobic conditions.

Jacob M. Weiner[a], Wei Hao Lee[b], Elizabeth M. Nolan[b], Amanda G. Oglesby[ac]

[a]Department of Pharmaceutical Sciences, School of Pharmacy, University of Maryland, Baltimore, Baltimore, Maryland, USA

[b]Department of Chemistry, Massachusetts Institute of Technology, Cambridge, MA, USA

[c]Department of Microbiology and Immunology, School of Medicine, University of Maryland, Baltimore, Baltimore, Maryland, USA

[#]Corresponding authors: Amanda Oglesby (aoglesby@rx.umaryland.edu) and Elizabeth Nolan (lnolan@mit.edu)

Table of contents

Table S1. Strains used in this study……………………………………………………………………………3

Table S2. Primers and probes used in this study…………………………………………………………………4-5

Table S3. Orbitrap high-resolution mass spectrometry data of purified pyochelin isolated from PAO1……………5

Figure S1. Anaerobic expression controls……………………………………………………………………………6

Figure S2. Effects of CP on metal inventory can only be seen in plastic vials……………………….....................7

Figure S3. Metal dropout conditions did not replicate response to CP………………………………………………..8

Figure S4. Anaerobic growth shifts the timing of sulfur metabolism and pyochelin biosynthesis gene expression……..9

Figure S5. *P. aeruginosa* only requires one of its heme uptake systems to benefit from the protective effects of heme……10

Figure S6. RT-PCR of predicted anaerobic PrrF targets did not show any PrrF regulation………………………..11

Figure S7. Aerobic cultures respond more to Zn withholding while anaerobic cultures respond more to Mn withholding…………………………………………………………………………………………………...12

Fig S8. Anaerobic CP treatment results in upregulation of genes encoding membrane remodeling proteins……12

Table S4. Transcripts significantly changed by CP treatment under anaerobic conditions………………Excel File

Table S5. Predicted PrrF targets that are significantly changed by CP under anaerobic conditions…… Excel File

Supporting references…………………………………………………………………………………………13

Table S1. Strains used in this study

Strain	Description	Reference
PAO1	Human wound isolate originally isolated in Australia	(1)
PA14	Clinical isolate from 1995 at the Massachusetts General Hospital, Boston, MA; virulent in a variety of plant and animal models of infection	(2)
PAO1Δ*prrF*	*prrF1,2* deletion in PAO1	(3)
PAO1 pUCP18	PAO1 carrying the pUCP18 empty vector	(4)
PAO1Δ*prrF* pUCP18	POA1Δ*prrF* carrying the pUCP18 empty vector	(4)
PAO1Δ*prrF* WT compliment (WT Comp)	PAO1Δ*prrF* carrying the entire *prrF* locus including 235 bp upstream of the *prrF1* start site, on the plasmid pUCP18	(4)
PAO1Δ*prrF* ΔH-IG complement (ΔH-IG comp)	PAO1Δ*prrF* carrying the *prrF* locus with a deletion of the PrrH intergenic region on the plasmid pUCP18	(4)
PAO1Δ*pvdA*	*pvdA* deletion in PAO1	(5)
PAO1Δ*pchEF*	*pcheF* deletion in PAO1	(5)
PAO1Δ*pvdA*Δ*pchEF*	*pvdA* and *pchEF* double deletion in PAO1	(5)
PAO1Δ*feoB*	*feoB* deletion in PAO1	(6)
PAO1Δ*hasR*	*hasR* deletion in PAO1	(7)
PAO1Δ*phuR*	*phuR* deletion in PAO1	(7)
PAO1Δ*hasR*ΔphuR	*hasR* and *phuR* double deletion in PAO1	(8)
PAO1/P_{antR}'*lacZ*	PAO1 with the P_{antR}-'*lacZ* reporter fusion containing the *antR* promotor and UTR fused to the *lacZ* coding region integrated at the chromosomal *att* site	(9)
PAO1Δ*prrF1,2*/P_{antR}'*lacZ*	PAO1Δ*prrF1,2* with the P_{antR}-'*lacZ* reporter fusion containing the *antR* promotor and UTR fused to the *lacZ* coding region integrated at the chromosomal *att* site	(9)

Table S2. Primers and probes used in this study

Name	Sequence (5' to 3')	Reference
16S Forward	GCGTAGGTGGTTCAGCAAGT	This study
16S Reverse	CATTTCACCGCTACACAGGA	This study
16S Probe	ACTGAGCTAGAGTACGGTAGAGGGTGGTGG	This study
pvdS Forward	CCTGGTCAACTTCATGATCCG	This study
pvdS Reverse	AGATGGGTGACGTTGTCGC	This study
pvdS Probe	CCTGGTGCACTGCCGCAAGGT	This study
lasA Forward	GCATTTCTCGCTGCTCTA	This study
lasA Reverse	GACAGTCGTTGTCGTAGTT	This study
lasA Probe	CGTACCGGATCAACGTCGGCA	This study
hemN Forward	CTTCTGCACCTCCATGCTGAA	This study
hemN Reverse	CTCAACCTGCTGGACGACGAT	This study
hemN Probe	TCCGCGAACTCGGTTTC	This study
arcA Forward	GTCCTCGAGATGCACAAT	This study
arcA Reverse	GTCGGCGGTGATCTTG	This study
arcA Probe	TGACCGAGACCATCCAGAACCCG	This study
napE Forward	CCAGGCTCTTCCTGTTTC	This study
napE Reverse	GCCAGTAGCTGGTACATC	This study
napE Probe	AGACGCAGAAACCGAAGCCGC	This study
nirS Forward	TACTTGGCGTCGAGGTTCTTC	This study
nirS Reverse	GAAAGTCGCCGAACTACAGGG	This study
nirS Probe	CGAGACCCTGGAACCGAAGCAGAT	This study
azu Forward	CGACCAGATGCAGTTCA	This study
azu Reverse	ACTGCTTGCAGCTCTT	This study
azu Probe	ACACCAATGCCATCACCGTCGAC	This study
pvdA Forward	CCGTATTCCTTCGTCAACTA	This study
pvdA Reverse	ATCCGGCAGGGATAGAA	This study
pvdA Probe	CACAAGCACGATCGCCTGGTCG	This study
phzA1 Forward	TTGCGAGAACCACTACATC	(10)
phzA1 Reverse	TTTGCGGAACGGCTATT	(10)
phzA1 Probe	ACCCGATGCAGAAATTGCGTGCAT	(10)
phzA Conserved Forward	ACAACGTGCGGATCTTC	(10)
phzA Conserved Reverse	TGTAGTGGTTCTCGCAATAG	(10)
phzA Conserved Probe	TCGCACTCGACCCAGAAGTGGTTC	(10)
prrF Forward	AACTGGTCGCGAGATCAGC	(4)
prrF Reverse	CCGTGATTAGCCTGATGAGGAG	(4)
prrF Probe	CCCACGCAGTCGGACTCTTCAGATT	(4)
pchE Forward	CTGGTGGACCTGCTATG	This study
pchE Reverse	CGAGATCGGCCAACAG	This study
pchE Probe	TGCGGCCTTGCTCGACGAC	This study
cysN Forward	GTGGTCGCCATCAACA	This study
cysN Reverse	GTCTTCAGACCGATCTTCTC	This study
cysN Probe	ATCAAGGCCGACTACCTGGCCT	This study
feoB Forward	CGCAGATCGGCTTGAT	This study
feoB Reverse	CGGCACGAACGACTT	This study
feoB Probe	ACCTGTTCCTCTCGCTGCTCGA	This study
hasR Forward	CTACCAGACCGACACCTA	This study
hasR Reverse	GAAGAACTCCAGGCCATAG	This study
hasR Probe	ACCTCGACCTTCGCTCTCGACG	This study
phuR Forward	GAACGACTGGACCTTCAC	This study
phuR Reverse	GTTCTGGCTCTGCTTCAT	This study
phuR Probe	CATGGAGCCGCACATCACCGAC	This study
prrH Forward	ATTCGGCCGGAGACGACCGTT	(4)

Name	Sequence (5' to 3')	Reference
prrH Reverse	CTGGCGATGGAATGAATGAGAACCG	(4)
prrH Probe	CGACCAGTTGGTGTAATAATAACTATT	(4)
znuA Forward	GGCTCGACGGGAAACTC	(10)
znuA Reverse	CGTAGGCCTCCTCGAAATAG	(10)
znuA Probe	CGGCAAGCCTTTCTTCGTCTTCCA	(10)
cntO Forward	TTGACAGCGCTCGTATC	(10)
cntO Reverse	AACTCCGAAGTGGTGAAG	(10)
cntO Probe	TGTACTCGAACATCGTCAGGCCGC	(10)
mntH1 Forward	CTGGGCGTCTCGATTAC	This Study
mntH1 Reverse	CAGTTCGACGAAGAAACAC	This Study
mntH1 Probe	TCACCGCCTTCGATACCCTGATCG	This Study
mntH2 Forward	CGTTGCTGACACTGATCT	This Study
mntH2 Reverse	GAATGCAGGTAGAGGTTGT	This Study
mntH2 Probe	CTTCGCGGTGTTGCTGCTCTC	This Study
sodA Forward	CGTCGGCTGCAACAA	This Study
sodA Reverse	ATGGCCCGGCTGATA	This Study
sodA Probe	CACCAGGGCCAGTTCCTCATCTG	This Study
sodB Forward	AACACCTACGTGGTGAACCTGA	(11)
sodB Reverse	TGACGATCTCTTCGAGGCTCTT	(11)
sodB Probe	CCTGATCCCGGGCACCGAGTT	(11)

Table S3. Orbitrap high-resolution mass spectrometry data of purified pyochelin[a] isolated from *P. aeruginosa* PAO1.

Retention time on analytical HPLC column (min)	High-resolution MS elution time (min)	Detected m/z value (assigned as $[M+H]^+$ ion)	Within 4 ppm of calculated mass?
Peak 1: 20.3 min	18.11	325.06689	Yes
	19.40	325.06705	Yes
Peak 2: 21.3 min	18.12	325.06714	Yes
	19.4	325.06717	Yes

[a] Calculated exact mass = 325.08607, $[M+H]^+$ ion.

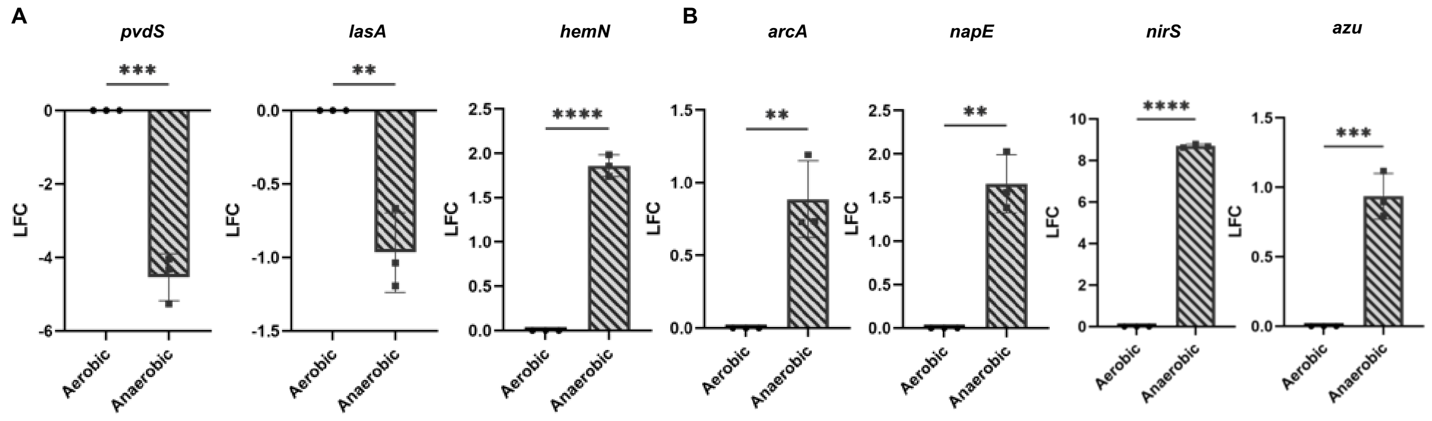

Figure S1. Anaerobic expression controls. RT-PCR expression analysis of genes shown to be (A) downregulated in anaerobic conditions (*pvdS* and *lasA*) and (B) upregulated under anaerobic conditions (*hemN*, *arcA*, *napE*, *nirS*, and *azu*) in cultures grown aerobically and anaerobically grown in metal replete CDM. Log_2 fold change (LFC) was determined in reference to aerobic cultures. Significance was determined using a Student's two-tailed t-test assuming unequal variances (n=3) *, P<0.05, **, P<0.01, ***, P<0.001, ****, P<0.0001.

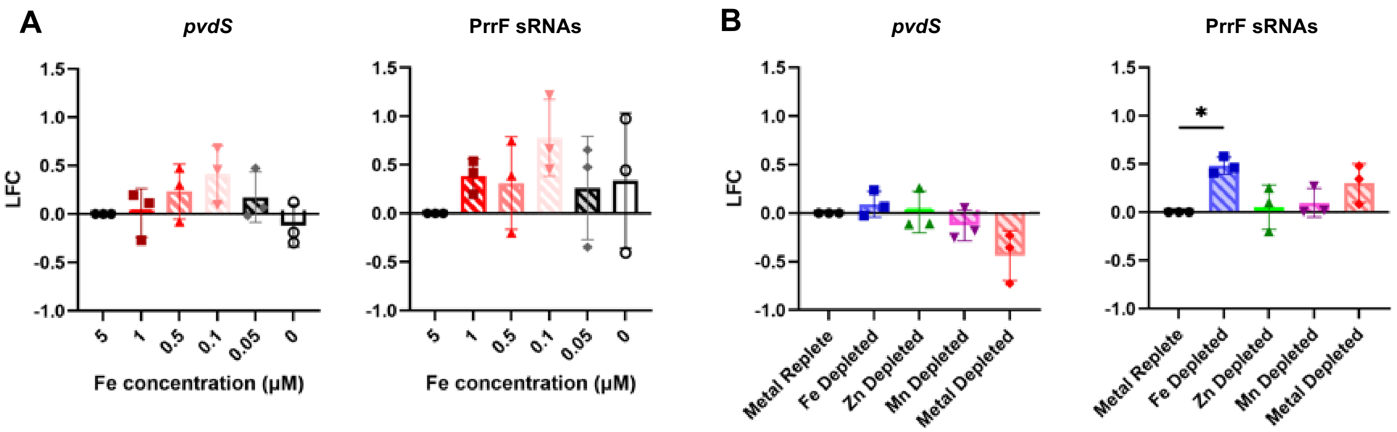

Figure S2. Metal dropout conditions did not replicate response to CP. (A) RT-PCR expression analysis of *pvdS* (left) and the PrrF sRNAs (right) in anaerobic cultures grown in metal replete CDM and CDM 1μM, 0.5μM, 0.1μM, 0.05μM, and 0μM iron. (B) RT-PCR expression analysis of *pvdS* (left) and the PrrF sRNAs (right) in anaerobic cultures grown in metal replete CDM, zinc depleted CDM, and iron/zinc depleted CDM. LFC was determined in reference to metal replete cultures. Significance was determined by one-way ANOVA with Tukey's multiple comparison test (n=3).*, P<0.05, **, P<0.01, ***, P<0.001, ****, P<0.0001.

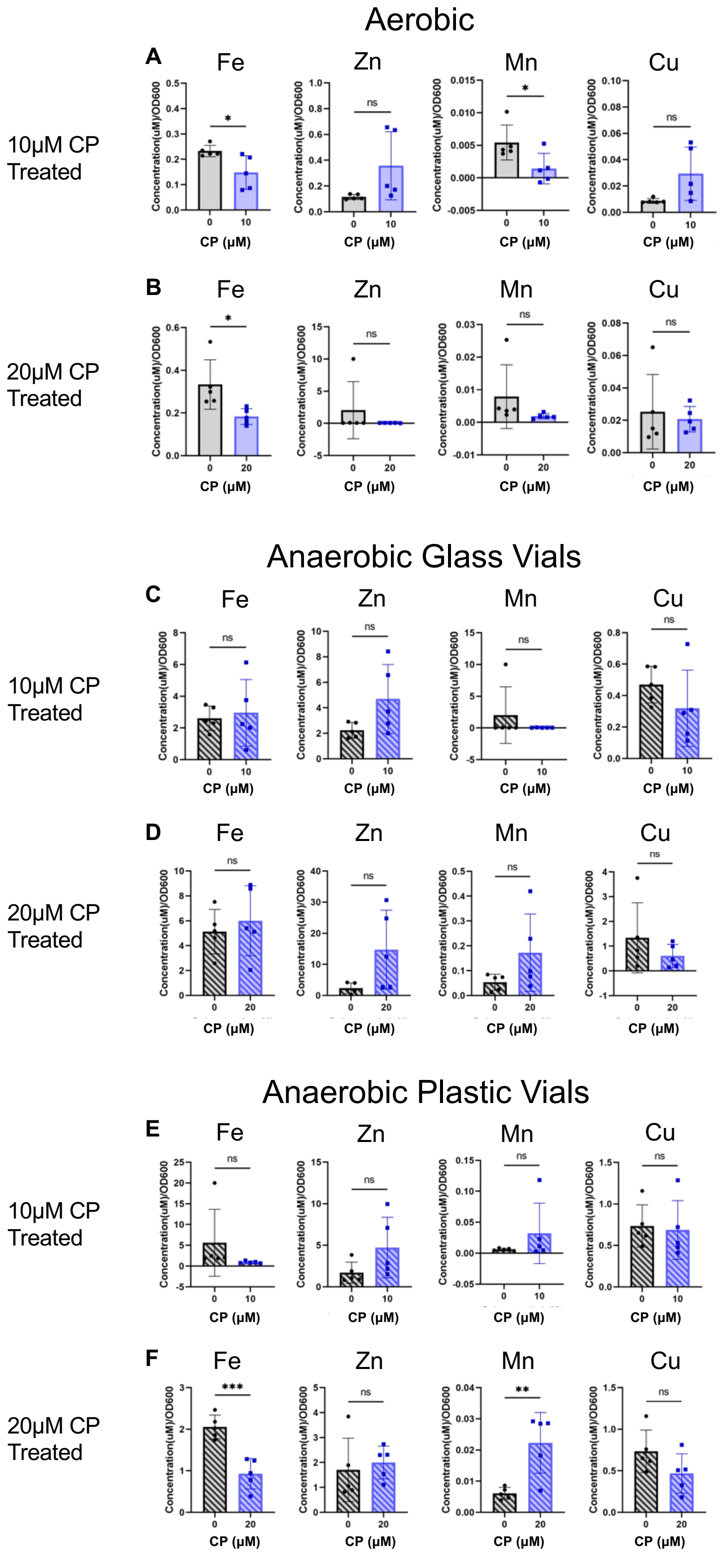

Figure S3. Effects of CP on metal inventory can only be seen when treated in plastic vials. ICP-MS analysis of metal inventories in aerobic cultures grown with metal replete CDM in the absence or presence of (A) 10µM CP or (B) 20µM CP. ICP-MS analysis of metal inventories in anaerobic cultures grown in glass vials with metal replete CDM in the absence or presence of (C) 10µM CP or (D) 20µM CP. ICP-MS analysis of metal inventories in anaerobic cultures grown in plastic vials with metal replete CDM in the absence or presence of (E) 10µM CP or (F) 20µM CP. Significance was determined using a Student's two-tailed t-test assuming unequal variances (n=5) *, $P<0.05$, **, $P<0.01$, ***, $P<0.001$, ****, $P<0.0001$.

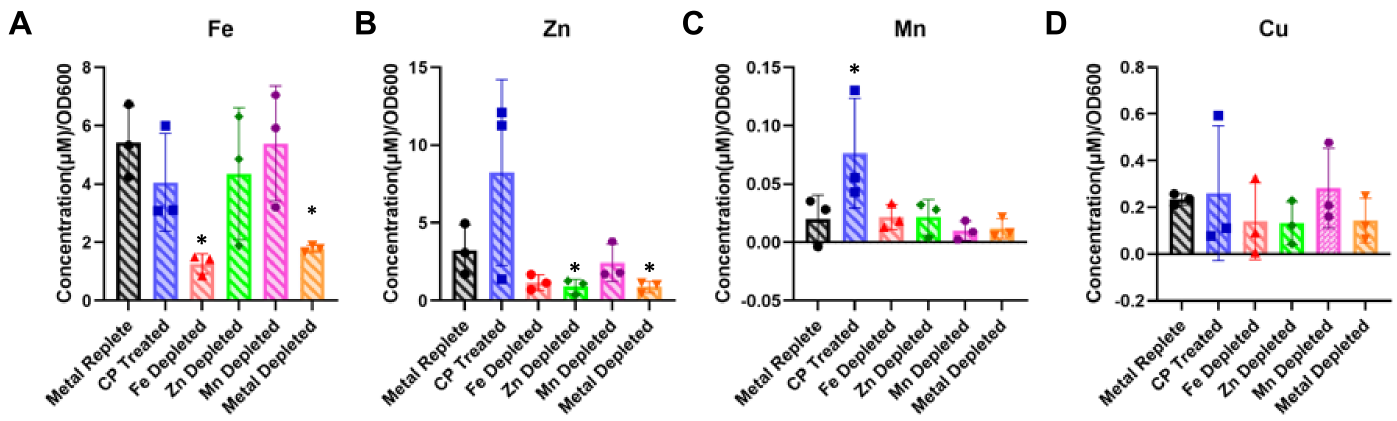

Figure S4. CP treatment has different effects on cell-associated metal inventories than metal depletion. ICP-MS analysis of cell-associated (A) iron, (B) zinc, (C) manganese, and (D) copper in anaerobic cultures grown in CDM under metal replete, CP treated, individual metal depletion, and multi-metal depletion conditions. Significance was determined by one-way ANOVA with Tukey's multiple comparison test (n=3): *, P<0.05; **, P<0.01; ***, P<0.001; ****, P<0.0001.

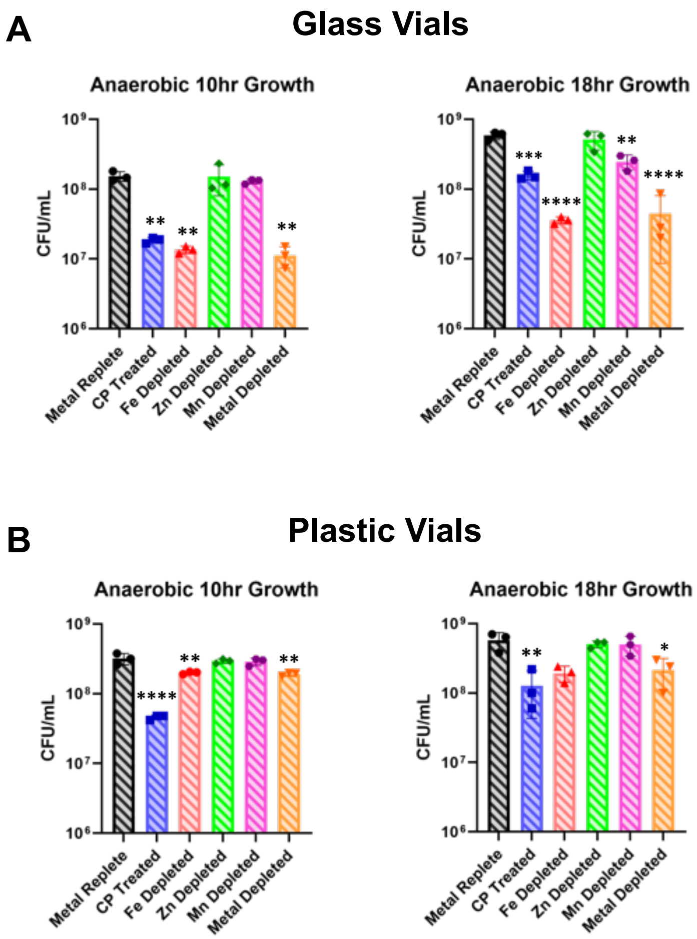

Figure S5. Anaerobic growth and response to metal depletion is different when cultured in glass or plastic serum vials. Growth of cultures grown in metal replete, 10µM CP treated, Fe-dropout, Zn-dropout, Mn-dropout, and metal depleted CDM under (C) Aerobic and (D) anaerobic conditions. Significance was determined by one-way ANOVA with Tukey's multiple comparison test (n=3): *, P<0.05; **, P<0.01; ***, P<0.001; ****, P<0.0001.

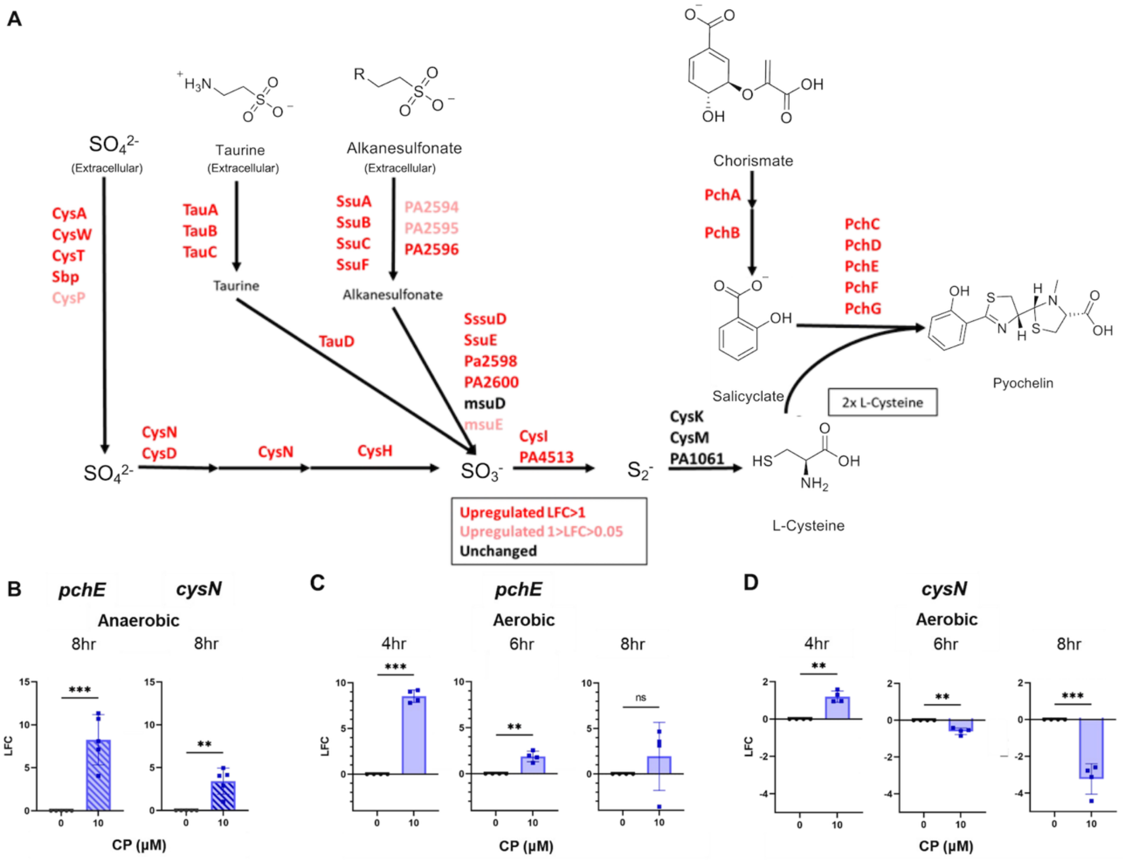

Figure S6. Anaerobic growth shifts the timing of sulfur metabolism and pyochelin biosynthesis gene expression. (A) Pathway analysis of sulfur metabolism and pyochelin biosynthesis. (B) RT-PCR analysis of *pchE* (left) and *cysN* (right) in anaerobic cultures grown for 8hrs in metal replete CDM in the presence or absence of 10μM CP. Time course RT-PCR analysis of (C) *pchE* and (D) cysN expression at 4hr (left), 6hr (middle), and 8hrs (right) in aerobic cultures grown in the same media conditions. LFC was determined in reference to untreated cultures. Significance was determined using a Student's two-tailed t-test assuming unequal variances (n=3) *, P<0.05, **, P<0.01, ***, P<0.001, ****, P<0.0001.

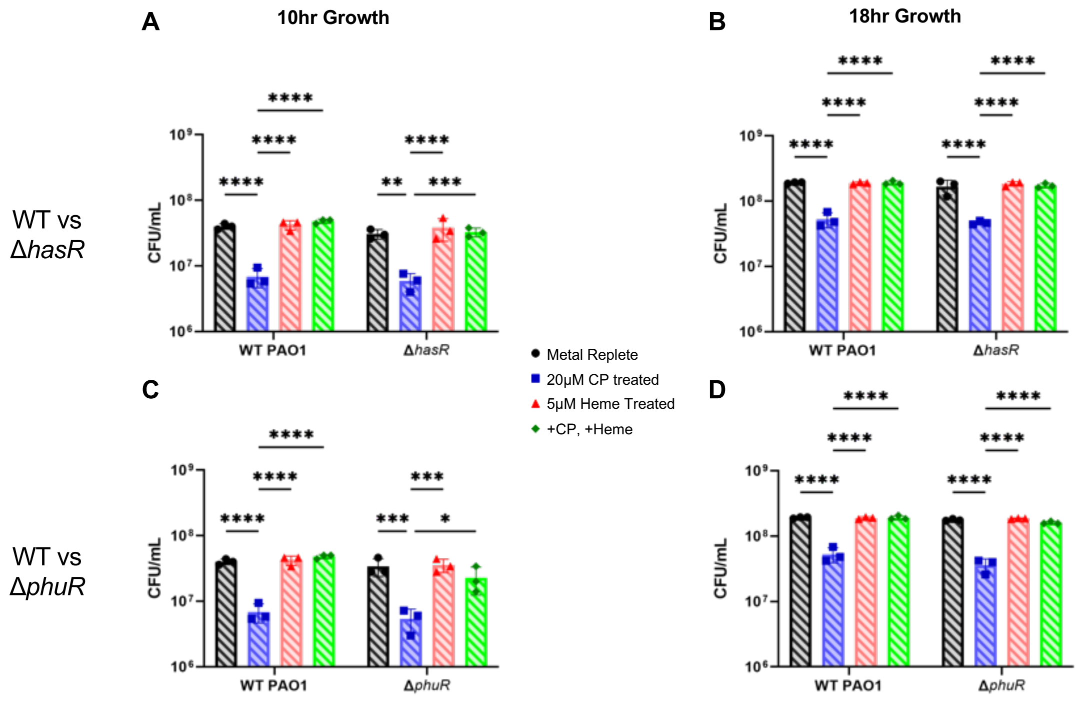

Figure S7. *P. aeruginosa* only requires one of its heme uptake systems to benefit from the protective effects of heme. Anaerobic growth of WT PAO1 and PAO1Δ*hasR* grown in metal replete CDM in the absence or presence of 20μM CP, 5μM heme, or a combination of 20μM CP and 5μM heme after (A) 10 hours and (B) 18 hours. Anaerobic growth of WT PAO1 and PAO1Δ*phuR* grown under the same conditions after (C) 10 hours and (D) 18 hours. Significance was determined by two-way ANOVA with Tukey's multiple comparison test (n=3). *, P<0.05, **, P<0.01, ***, P<0.001, ****, P<0.0001.

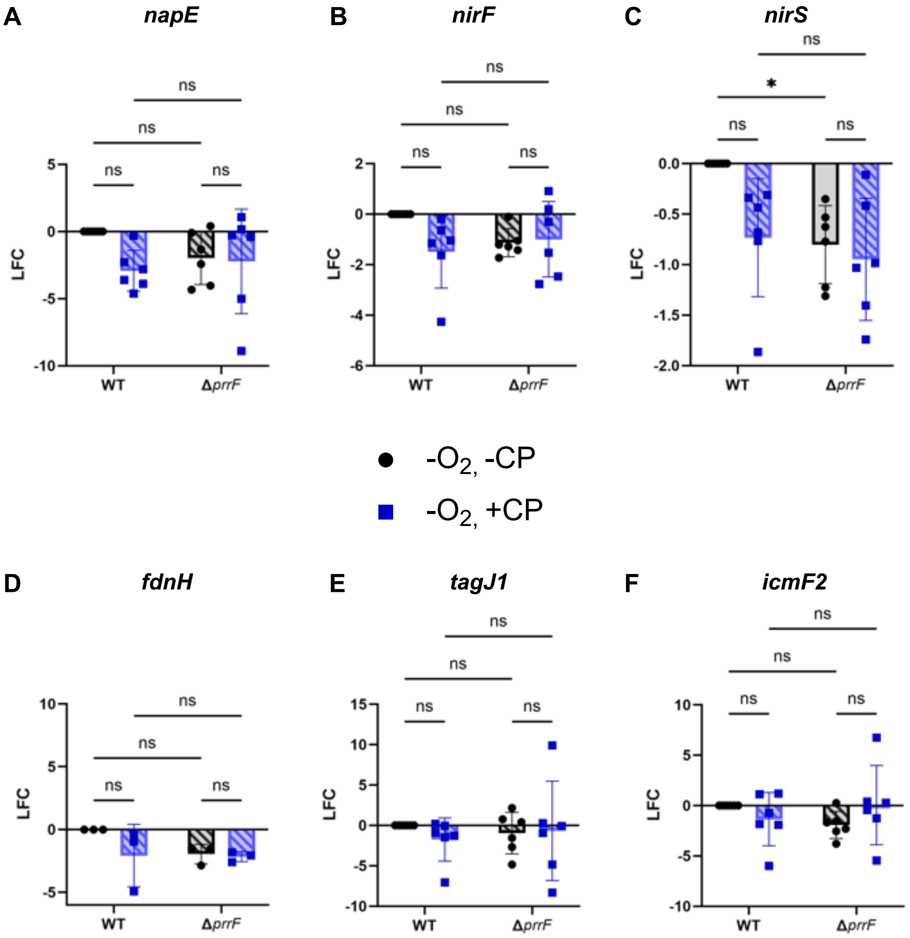

Figure S8. RT-PCR of predicted anaerobic PrrF targets did not show any PrrF regulation. Gene expression of (A) *napE* (B) *nirF* (C) *nirS* (D) *fdnH* (E) *tagJ1* and (F) *icmF2* in cultures of PAO1 and PAO1Δ*prrF* grown anaerobically in metal replete CDM in the absence or presence of 10μM CP measured via RT-PCR. LFC was determined in reference to untreated WT cultures. Significance was determined by one-way ANOVA with Tukey's multiple comparison test (n=3-6). *, P<0.05, **, P<0.01, ***, P<0.001, ****, P<0.0001.

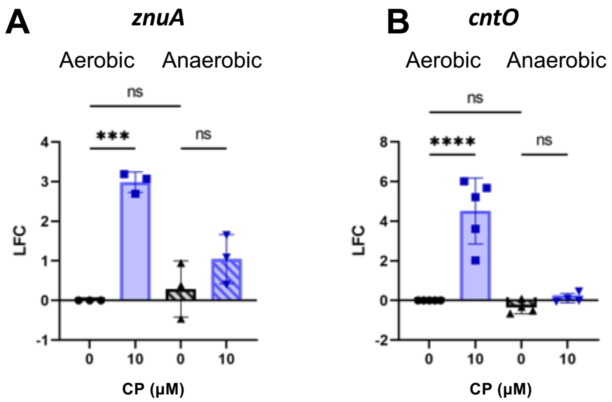

Figure S9. Aerobic cultures respond more to Zn withholding while anaerobic cultures respond more to Mn withholding. Gene expression of (A) *znuA* and (B) *cntO* in cultures grown in metal replete CDM in the presence or absence of 10μM CP. LFC was determined in reference to untreated aerobic cultures. Significance was determined by one-way ANOVA with Tukey's multiple comparison test (n=3-5). *, P<0.05, **, P<0.01, ***, P<0.001, ****, P<0.0001.

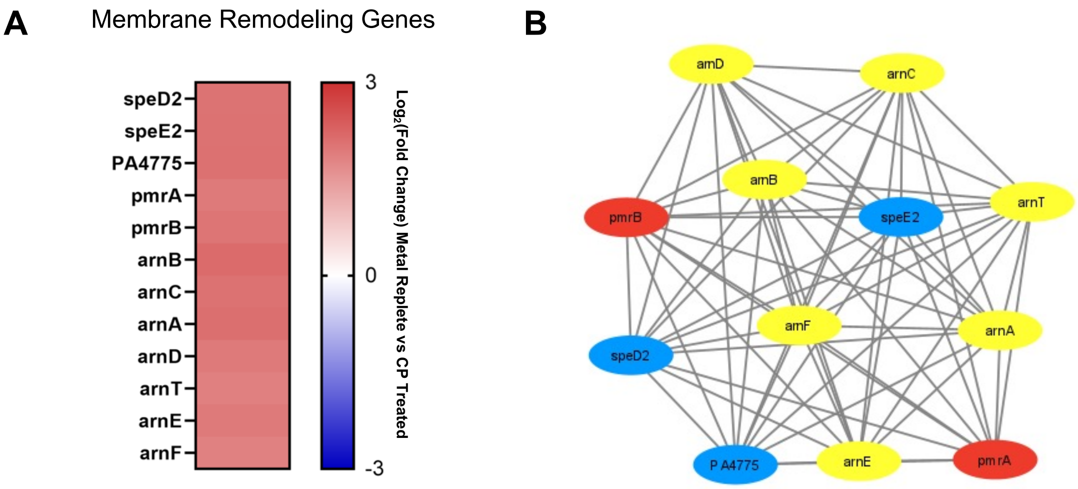

Figure S10. Anaerobic CP treatment results in upregulation of genes encoding membrane remodeling proteins. (A) Heat map displaying expression of genes involved in membrane remodeling. (B) network analysis of genes involved in membrane remodeling showing genes involved in lipid A biosynthesis (yellow) and spermidine biosynthesis (blue) and for the PmrAB two component system (red).

Supplementary References

1. Holloway BW. 1955. Genetic Recombination in *Pseudomonas aeruginosa*. Microbiology. Microbiology Society.

2. Rahme LG, Stevens EJ, Wolfort SF, Shao J, Tompkins RG, Ausubel FM. 1995. Common virulence factors for bacterial pathogenicity in plants and animals. Science 268:1899–1902.

3. Wilderman PJ, Sowa NA, FitzGerald DJ, FitzGerald PC, Gottesman S, Ochsner UA, Vasil ML. 2004. Identification of tandem duplicate regulatory small RNAs in *Pseudomonas aeruginosa* involved in iron homeostasis. Proc Natl Acad Sci U S A 101:9792–9797.

4. Hoang T-M, Huang W, Gans J, Weiner J, Nowak E, Barbier M, Wilks A, Kane MA, Oglesby AG. 2023. The heme-responsive PrrH sRNA regulates *Pseudomonas aeruginosa* pyochelin gene expression. mSphere 8:e00392-23.

5. Nguyen AT, O’Neill MJ, Watts AM, Robson CL, Lamont IL, Wilks A, Oglesby-Sherrouse AG. 2014. Adaptation of iron homeostasis pathways by a *Pseudomonas aeruginosa* pyoverdine mutant in the cystic fibrosis lung. J Bacteriol 196:2265–2276.

6. Oglesby-Sherrouse AG, Djapgne L, Nguyen AT, Vasil AI, Vasil ML. 2014. The complex interplay of iron, biofilm formation, and mucoidy affecting antimicrobial resistance of *Pseudomonas aeruginosa*. Pathog Dis 70:307–320.

7. Smith AD, Wilks A. 2015. Differential contributions of the outer membrane receptors PhuR and HasR to heme acquisition in *Pseudomonas aeruginosa*. J Biol Chem 290:7756–7766.

8. Minandri F, Imperi F, Frangipani E, Bonchi C, Visaggio D, Facchini M, Pasquali P, Bragonzi A, Visca P. 2016. Role of Iron Uptake Systems in *Pseudomonas aeruginosa* Virulence and Airway Infection. Infect Immun 84:2324–2335.

9. Djapgne L, Panja S, Brewer LK, Gans JH, Kane MA, Woodson SA, Oglesby-Sherrouse AG. 2018. The *Pseudomonas aeruginosa* PrrF1 and PrrF2 Small Regulatory RNAs Promote 2-Alkyl-4-Quinolone

Production through Redundant Regulation of the *antR* mRNA. Journal of Bacteriology 200:10.1128/jb.00704-17.

10. Nelson CE, Huang W, Brewer LK, Nguyen AT, Kane MA, Wilks A, Oglesby-Sherrouse AG. 2019. Proteomic Analysis of the *Pseudomonas aeruginosa* Iron Starvation Response Reveals PrrF Small Regulatory RNA-Dependent Iron Regulation of Twitching Motility, Amino Acid Metabolism, and Zinc Homeostasis Proteins. Journal of Bacteriology 201:10.1128/jb.00754-18.

11. Reinhart AA, Nguyen AT, Brewer LK, Bevere J, Jones JW, Kane MA, Damron FH, Barbier M, Oglesby-Sherrouse AG. 2017. The *Pseudomonas aeruginosa* PrrF Small RNAs Regulate Iron Homeostasis during Acute Murine Lung Infection. Infection and Immunity 85:10.1128/iai.00764-16.
